# Catalytic efficiency of GO-PANI nanocomposite in the synthesis of N-Aryl-1,4-Dihydropyridine and hydroquinoline derivatives

**DOI:** 10.1038/s41598-024-82907-5

**Published:** 2025-01-22

**Authors:** Hossein Ghafuri, Moghadaseh keshvari, Fatemeh Eshrati, Peyman Hanifehnejad, Atefeh Emami, Hamid Reza Esmaili Zand

**Affiliations:** Catalysts and Organic Synthesis Research Laboratory, Department of Chemistry, University of Science and Technology, 16846-13114 Tehran, Iran

**Keywords:** Graphene Oxide (GO), Polyaniline (PANI), Heterogeneous Nanocatalyst, 1,4-Dihydropyridine(1,4-DHP), Hydroquinoline, Green chemistry, Catalysis, Catalyst synthesis, Catalytic mechanisms, Heterogeneous catalysis

## Abstract

In this research, graphene oxide-polyaniline (GO-PANI) nanocomposite was successfully synthesized and its catalytic performance was evaluated for the synthesis of N-aryl-1,4-dihydropyridine (1,4-DHP) and hydroquinoline derivatives. The GO nanosheets were prepared using the Hummers’ method, and in-situ polymerization of aniline was conducted with ammonium persulfate (APS) serving as the polymerization initiator. The synthesized nanocomposite demonstrated notable efficiency, achieving yields of 80–94% for 1,4-DHP derivatives and 84–96% for hydroquinoline derivatives. The GO-PANI nanocomposite was thoroughly characterized by various techniques, including Fourier Transforms Infrared spectroscopy (FT-IR), Field Emission Scanning Electron Microscopy (FE-SEM), X-ray Diffraction analysis (XRD), Thermogravimetric analysis (TGA), and Energy Dispersive X-ray spectroscopy (EDS), all of which confirmed the successful synthesis of the nanocomposite. Furthermore, after ten cycles of reusability testing, the nanocomposite retained its high catalytic performance with no significant degradation. This findings indicate that the GO-PANI nanocomposite is a promising non-metal catalyst for the synthesis of N-aryl-1,4-dihydropyridine and hydroquinoline derivatives.

## Introduction

Carbon-based materials, including carbon nanotubes, fullerenes, and activated carbon, have attracted considerable interest owing to their high surface area, mechanical strength, and thermal stability^1^. However, each of these materials presents specific limitations: carbon nanotubes frequently encounter challenges related to dispersion and aggregation, fullerenes are associated with complex synthesis processes and scalability challenges, and activated carbon suffers from insufficient conductivity and mechanical strength for more advanced applications^2^.

Graphene, another carbon-based material, has been acknowledged for its potential to overcome several of these limitations, owing to its low production costs, high surface area, mechanical and thermal stability, and excellent conductivity^3–5^. However, the limited diversity of chemical functional groups in graphene constrains its versatility. Graphene oxide (GO), an oxidized derivative of graphene, mitigates this limitation by incorporating various oxygen-containing functional groups, thereby enhancing its applicability across a broader range of uses^6–8^.

The functional groups in GO enable it to form bonds with various materials, including metals, ceramics, and polymers, thus facilitating the development of GO-based nanocomposites^9^. When combined with polymers such as polyethylene, polystyrene, and polyaniline (PANI), GO significantly enhances the properties of these materials^10^. For example, polyethylene-GO composites exhibit improved mechanical strength and thermal stability, while polystyrene-GO composites demonstrate increased durability and resistance to environmental degradation^11^. Among these polymers, PANI is particularly notable for its high electrochemical activity, ease of synthesis, and excellent conductivity^12–15^.

Recent studies have emphasized the exceptional chemical and thermal stability of GO-PANI nanocomposites, along with the impressive electrochemical and mechanical properties, making them highly valuable in a wide range of applications. These applications include biosensors, where GO-PANI nanocomposites enhance sensitivity and selectivity^16,17^, wound dressing materials that benefit from their antibacterial properties and biocompatibility, scaffolds for bone regeneration and tissue engineering due to their mechanical strength and biocompatibility, and catalysis^18^, where their high surface area and conductivity contribute to improved catalytic performance.

Catalysis is a fundamental process in the synthesis of a wide range of chemical compounds, including pharmaceuticals. Multi-component reactions (MCRs) are particularly effective in synthesizing heterocyclic compounds due to their operational simplicity, high atom economy, and minimal by-product generation^19–21^. These reactions enable the formation of complex molecules in a single step by combining three or more reactants, thereby offering a streamlined and efficient synthetic approach. MCRs are particularly advantageous for the synthesis of medicinal heterocyclic compounds, such as N-aryl-1,4-dihydropyridine and hydroquinoline derivatives, which exhibit a range of pharmacological activities, including blood pressure reduction, calcium channel blocking, and vasodilation^22–24^. These compounds are essential in the development of antimicrobial agents, cardiovascular therapies, and other pharmaceutical applications^25^.

This research aims to synthesize a composite of graphene oxide and polyaniline and to evaluate its effectiveness as a metal-free catalyst for the synthesis of N-aryl-1,4-dihydropyridine and hydroquinoline derivatives. The catalytic activity of the GO-PANI nanocomposite will be assessed in terms of yield, selectivity, and reaction time. This study seeks to demonstrate the potential of the GO-PANI nanocomposite as an efficient and environmentally friendly catalyst for the synthesis of heterocyclic pharmaceutical compounds.

### Experimental

#### Instrumentation

In this research, chemicals and solvents were sourced from reputable companies, including Sigma-Aldrich and Merck. Various instruments were utilized to acquire the necessary data. FT-IR spectra were recorded using a Shimadzu IR-470 spectrometer (Japan) with the KBr pellet sampling method. NMR spectra were obtained on a Bruker DRX-500 Avance spectrometer (Germany). XRD patterns were generated using a JEOL JDX–8030 diffractometer (Japan), and EDS analysis was conducted with a Numerix DXP–X10P system (USA). FE-SEM images were captured with a Sigma Zeiss instrument (Germany), and BET surface area analysis was performed using a Micromeritics ASAP 2020 instrument (USA). Raman spectra were recorded with a Raman Ram-532-004 instrument (China). Melting points were determined using an Electrothermal 9100 apparatus (UK). Additionally, TGA analysis was performed using a TGA L001 instrument (USA) under a nitrogen atmosphere with a heating rate of 10 °C/min. All compounds were isolated through crystallization.

### Preparation of GO-PANI

According to our previous research, GO was synthesized using the modified Hummers’ method^26^. Specifically, a round-bottom flask containing 95 mL of H₂SO₄ (98%) as a highly acidic medium was used to combine 4 g of graphite and 2 g of NaNO_2_ as a stabilizing agent to facilitate the oxidation process. The mixture was sonicated at 65 $\:^\circ\:$C for 30 min and subsequently stirred for 1 h at 20 °C. Following this, 12 g of KMnO_4_ as the oxidant was gradually added to the mixture under sonication. The resulting mixture was then sonicated at 80 $\:^\circ\:$C for 30 min and stirred for an additional 1 h at 95 $\:^\circ\:$C. To this mixture, 250 mL of deionized (DI) water was added and heated at 100 $\:^\circ\:$C for 1 h, resulting in the formation of a brown mixture. The mixture was then poured into 450 mL of DI water, and H_2_O_2_ 5% was added drop by drop to produce a yellow-brown solution. The pH of the solution was adjusted to 6 by incorporating HCl 5%, followed by stirring the mixture for an additional 10 min at room temperature before filtration. To confirm the removal of manganese and Cl^–^, 5 mL of H_2_O_2_ 30% and 0.1 mL of AgNO_3_ 0.1 M were added to the filtrate solution. The resulting dark brown powder was then dried in a vacuum oven at 60 $\:^\circ\:$C for 24 h to achieve the desired morphology.

**Fig. 1:**
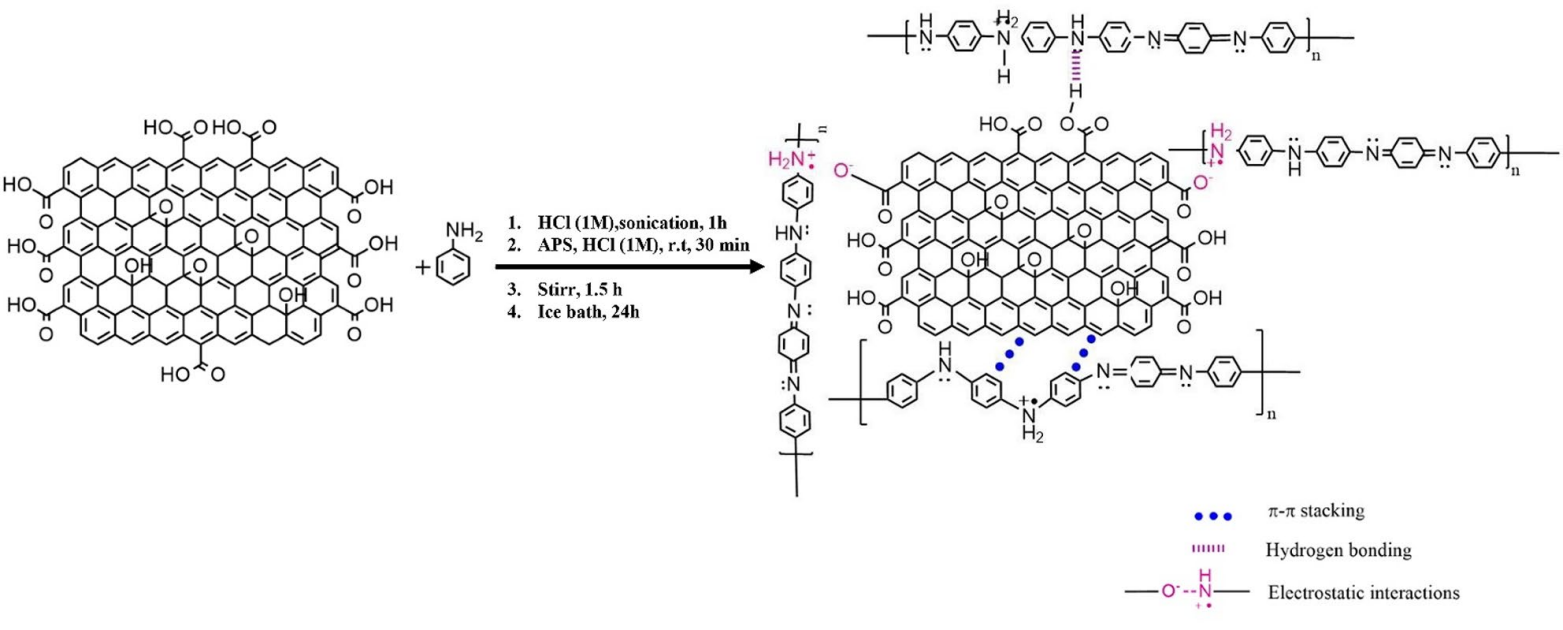
The schematic of modification of GO with PANI.

For the synthesis of the GO-PANI nanocomposite, GO was sonicated with 70 mL of HCl (1 M) for 1 h. The use of HCl aids in the dispersion of GO sheets and improves their interaction with aniline during the subsequent polymerization process. Following this, 2 g of GO and 4 g of aniline (maintaining a weight ratio of GO: aniline, 1:2) were added to the GO suspension and vigorously stirred for 2 h until the GO-aniline mixture was synthesized. Subsequently, a solution of 4.564 g of ammonium persulfate in 30 mL of HCl (1 M) was stirred at room temperature for 30 min. This solution was gradually added to the GO-aniline mixture and stirred for 1.5 h to ensure complete combination before initiating the polymerization. The mixture was then placed in an ice bath for 24 h to facilitate polymerization. Finally, the mixture was filtered, washed with deionized water, and dried in a vacuum oven at 60 $\:^\circ\:$C for 72 h to obtain the GO-PANI nanocomposite. The schematic representation of the GO-PANI nanocomposite synthesis via in-situ polymerization method is provided in Figs. 1^27–29^.

### General procedure for the synthesis of N-aryl-1,4-dihydropyridine derivatives

To a round-bottom flask, benzaldehyde (1 mmol), malononitrile or ethyl cyanoacetate (1 mmol), dimethyl acetylenedicarboxylate (DMAD) (1 mmol), aniline (1 mmol), 3 ml EtOH as the solvent, and 30 mg of the catalyst were added. The mixture was stirred at room temperature for 15 min. The progress of the reaction was monitored by thin-layer chromatography (TLC). Upon completion, the catalyst was separated via simple filtration using filter paper, and the final product was obtained through crystallization. The separated catalyst was washed with ethanol, specifically, 10–20 mL of ethanol was poured over the catalyst on the filter paper. This washing step was repeated 2–3 times to ensure the complete removal of the soluble product from the catalyst surface. After washing, the catalyst was dried under vacuum at 60 °C for 12 h, allowing it to be reused in subsequent reactions without significant loss of catalytic activity^30^.

### General procedure for the synthesis of hydroquinoline derivatives

To a round-bottom flask, 3-nitrobenzaldehyde (1 mmol), malononitrile (1 mmol), dimedone (1 mmol), methylaniline (1 mmol), 3 ml EtOH as the solvent, and 30 mg of the catalyst were added. The mixture was stirred at room temperature for 15 min. The progress of the reaction was monitored by thin-layer chromatography (TLC). Upon completion, the catalyst was separated via simple filtration using filter paper, and the final product was obtained through crystallization. The separated catalyst was washed with ethanol, specifically, 10–20 mL of ethanol was poured over the catalyst on the filter paper. This washing step was repeated 2–3 times to ensure the complete removal of the soluble product from the catalyst surface. After washing, the catalyst was dried under vacuum at 60 °C for 12 h, allowing it to be reused in subsequent reactions without significant loss of catalytic activity^31^.

### Selected spectra data

dimethyl 6-amino-5-cyano-4-(3-nitrophenyl)-1-(p-tolyl)-1,4-dihydropyridine-2,3-dicarboxylate (1a).

FTIR (KBr, cm^− 1^): 3444, 3332, 3072, 2983, 2181, 1747, 1568, 1348, 1112. The ¹H-NMR spectrum shows a singlet at δ 4.79 ppm, corresponding to the proton attached to the chiral carbon (CH). The broad singlet at δ 4.16 ppm is assigned to the amino group (NH₂). The aromatic protons are observed as multiplets between δ 7.21–7.31 ppm. Additionally, the methoxy groups (OMe) appear as singlets at δ 3.58 and 3.46 ppm, and the methyl group (Me) of the p-tolyl group resonates at δ 2.42 ppm. These assignments confirm the proposed structure of 1a. The detailed spectra and analysis images, including FTIR, NMR, and other relevant characterizations, are provided in the Supporting Information file.

2-amino-7,7-dimethyl-1-(4-methylphenyl)-4-(3-nitrophenyl)-5-oxo-1,4,5,6,7,8-hexahydroquinoline-3-carbonitrile (5b).

FTIR (KBr, cm^− 1^): 3481, 3382, 3072, 2960, 2177, 1693, 1529, 1348. The ¹H-NMR spectrum reveals a singlet at δ 4.81 ppm corresponding to the NH₂ group, while the chiral proton (CH) appears as a singlet at δ 4.17 ppm. The methyl protons resonate at δ 0.99 and 0.85 ppm. The multiplet in the aromatic region (δ 7.24–8.21 ppm) confirms the presence of the substituted aromatic rings. These data strongly support the proposed structure of 5b. The detailed spectra and analysis images, including FTIR, NMR, and other relevant characterizations, are provided in the Supporting Information file.

## Results and discussion

### Synthesizing GO-PANI nanocomposite

In this study, we successfully applied polyaniline onto the surface of graphene oxide by forming hydrogen bonds between the oxygen-containing groups of graphene oxide and the nitrogen atoms in polyaniline. Due to the availability of free electron pairs, the nitrogen atoms in polyaniline can form hydrogen bonds with other substances, thereby enhancing its functionality as a catalyst in multicomponent reactions. Consequently, polyaniline demonstrates significant potential as a catalyst for a variety of chemical reactions.

### Characterization of GO-PANI nanocomposite

**Fig. 2:**
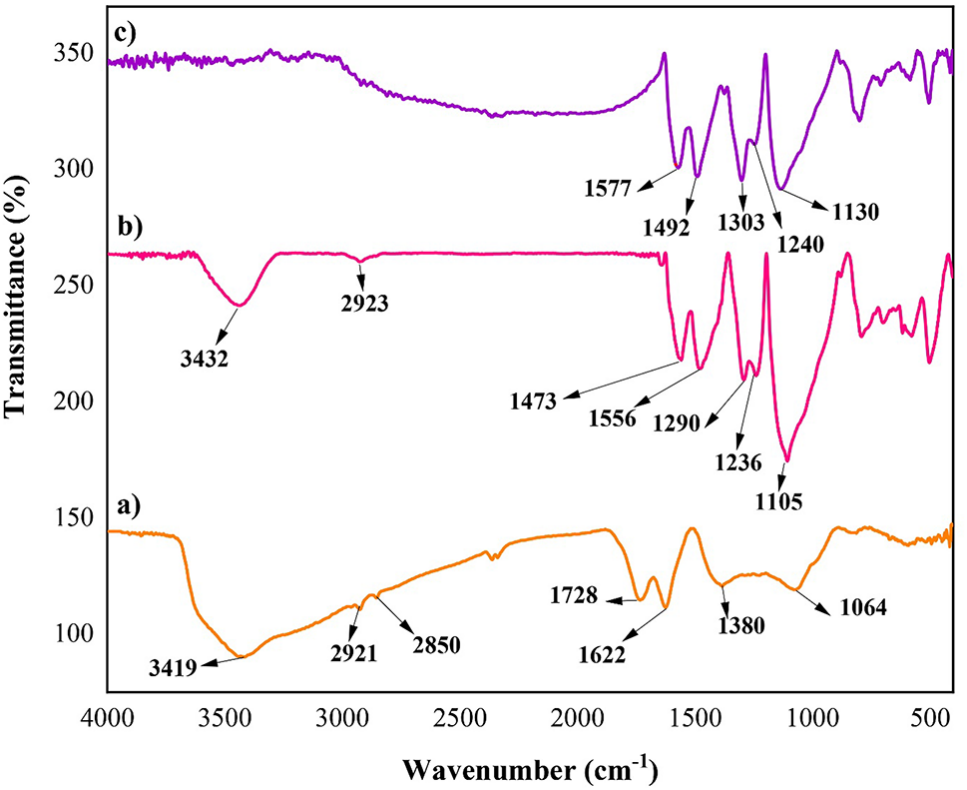
FT-IR spectra of (**a**) GO, (**b**) PANI, and (**c**) GO-PANI.

The functional groups of GO (Fig. 2-a), PANI (Fig. 2-b), and the GO-PANI nanocomposite (Fig. 2-c) were investigated by FT-IR spectroscopy. In Fig. 2-a, a broad peak around 3419 cm^− 1^ related to the stretching vibration of -OH and -COOH groups. The weak peaks observed around 2921 and 2850 cm^− 1^ are attributed to the stretching vibration of C-H groups. The stretching vibrations of C = O and C = C groups are present at approximately1728 and 1622 cm^− 1^, respectively. Additionally, the C-O and alkoxy stretching vibration groups are detected at around 1380 and 1064 cm^− 1^, respectively.

In Fig. 2-b, the characteristic peaks of PANI are observed at 3432, 2923, 1556, 1473, 1290, 1236, and 1105 cm^− 1^, consistent with the reported literature^32^.

Figure 2-c displays characteristic peaks corresponding to both GO and PANI, confirming the successful synthesis of the GO-PANI nanocomposite^33^. However, some shifts are observed, as the peaks at 1290, 1236, and 1105 cm⁻¹ in PANI shift to 1299, 1244, and 1128 cm⁻¹ in the GO-PANI nanocomposite. These shifts can be explained by the interactions between the GO nanosheets and PANI chains, which restrict the vibrations within the PANI structure^34^.

Additionally, the band at approximately 1303 cm^−1^, associated with C–N stretching in the quinoid ring, and the band at 1240 cm^−1^, corresponding to C–H bending vibrations, indicate the doped state of PANI in the GO-PANI nanocomposite. The presence of these bands confirms that PANI retains its electrochemical activity in the composite form. The stretching vibration at 1728 cm⁻¹, typically associated with the C = O groups in GO, is not observed in the FTIR spectrum of the GO-PANI nanocomposite. This may be attributed to the partial interaction or chemical bonding between the oxygen-containing groups of GO and the aniline monomers during polymerization. However, the overall structure of GO remains largely intact, as evidenced by the FTIR spectrum, where the characteristic bands of GO are still present. This indicates that while some functional groups may interact with PANI, the core structure of GO is preserved^33^.

**Fig. 3:**
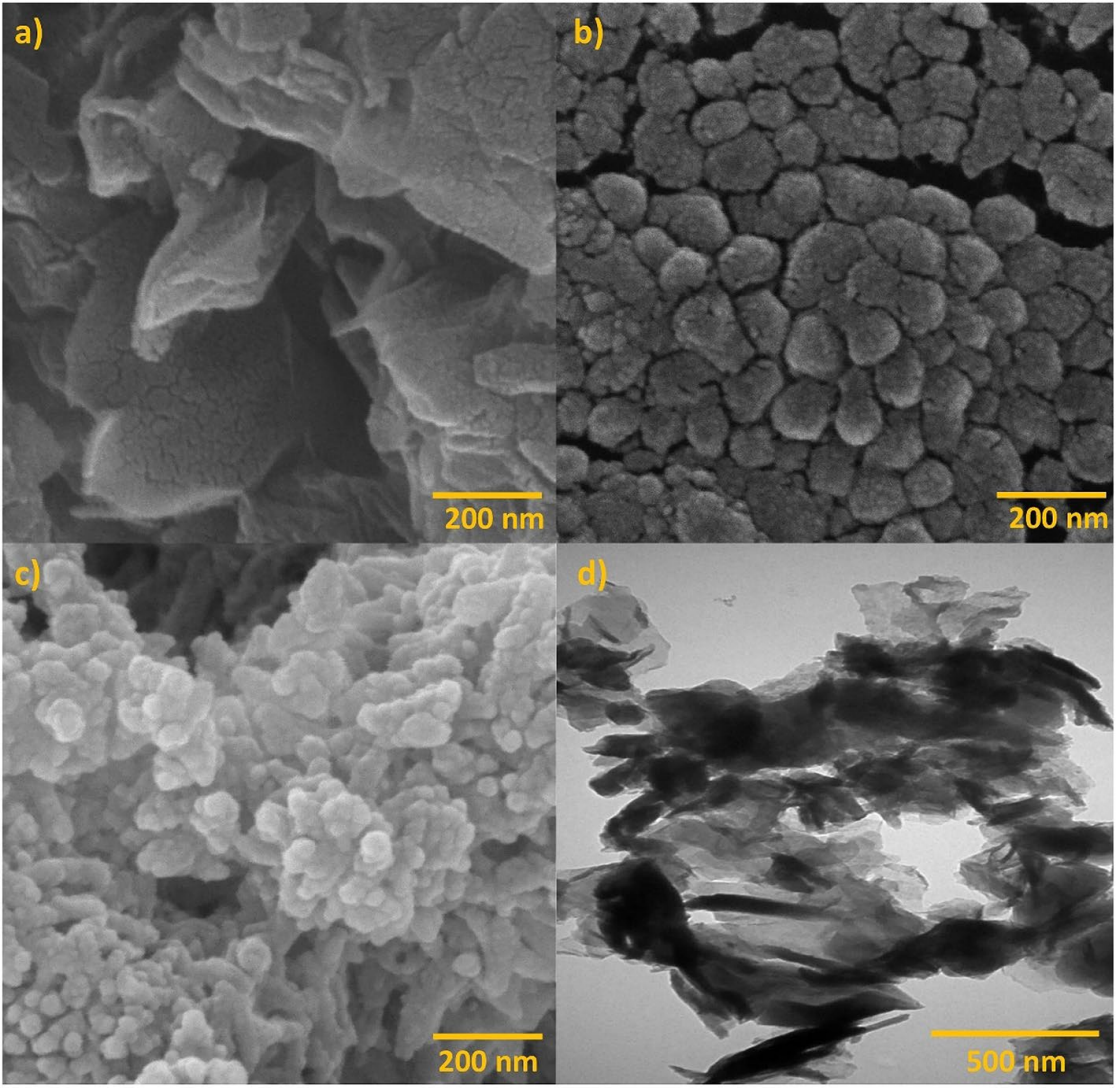
FE-SEM images of (**a**) GO, (**b**) PANI, (**c**) GO/PANI nanocomposite, and TEM image of (**d**) GO/PANI nanocomposite.

The morphology of GO, PANI, and the GO/PANI nanocomposite was thoroughly analyzed using FE-SEM, as shown in Fig. 3. Additionally, the TEM image of the GO/PANI nanocomposite was investigated and is also presented in Fig. 3.

The FE-SEM image of GO (Fig. 3-a) exhibits a characteristic layered and wrinkled morphology. The image shows thin, crumpled sheets that are stacked loosely together. The wrinkled structure of GO is indicative of its two-dimensional nature, with the layers loosely aggregated, forming a network of overlapping sheets. These wrinkles and folds contribute to the large surface area and potential for functionalization, which are critical for applications in areas such as energy storage, sensors, and catalysis.

The FE-SEM image of PANI (Fig. 3-b) reveals a distinctly different morphology compared to GO. PANI appears as a collection of spherical or granular particles, closely packed together. This granular structure is common for polyaniline and is indicative of its semi-crystalline nature. The particles are aggregated, but the surface appears to be relatively smooth, suggesting that the PANI particles are densely packed.

In the FE-SEM image of the GO/PANI nanocomposite (Fig. 3-c), the morphology reflects a combination of the features observed in the individual components. The image shows that the PANI particles are distributed over the surface of the GO sheets. The granular structure of PANI is visible, but it is integrated with the wrinkled GO sheets. This integration suggests strong interactions between the GO and PANI, likely due to the intercalation of PANI into the GO layers or its deposition onto the GO surface. The composite structure maintains the high surface area characteristic of GO while incorporating the conductive properties of PANI, making the material suitable for applications requiring high surface area.

The TEM image of the GO/PANI nanocomposite (Fig. 3-d) provides further insight into the composite’s microstructure. The image shows thin, transparent sheets of GO, with PANI likely dispersed or intercalated within the GO layers. The transparency of the GO sheets in the TEM image indicates their thin nature, and the darker regions suggest areas where PANI may be interacting with the GO. This interaction likely enhances the composite’s structural stability properties, as the PANI could act as a spacer, preventing the restacking of GO sheets and maintaining a high surface area^35,36^.

**Fig. 4:**
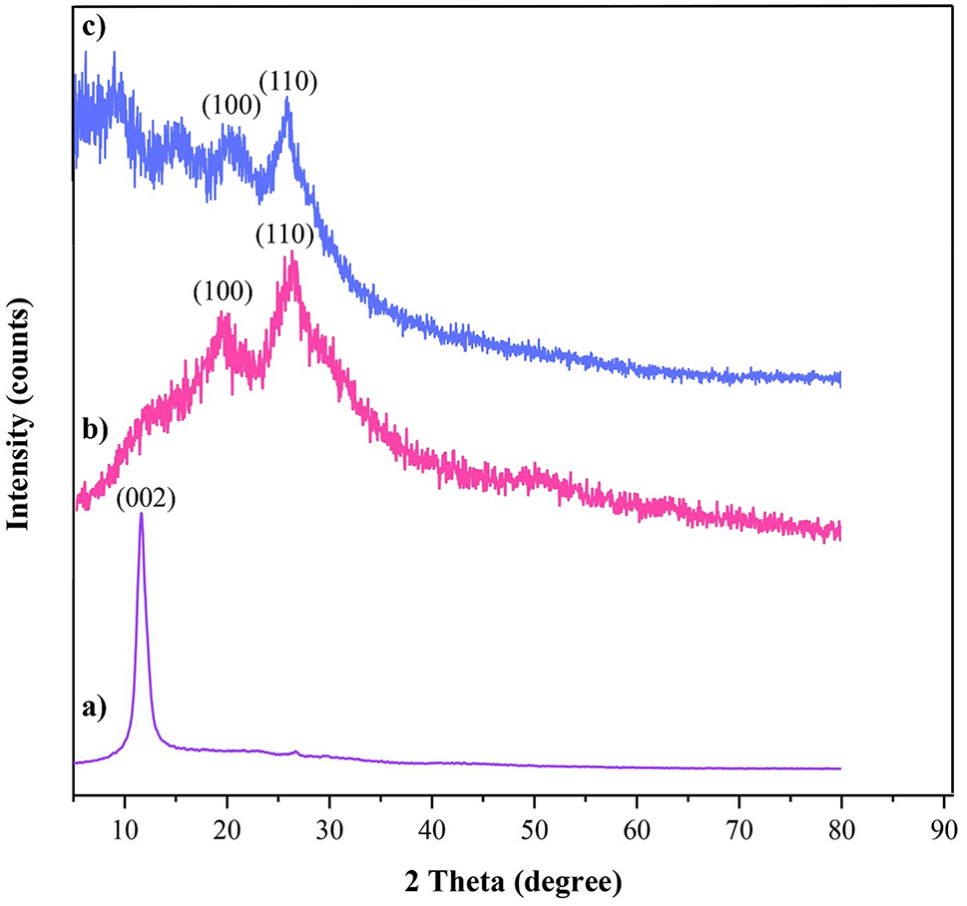
XRD of (**a**) GO, (**b**) PANI, and (**c**) GO-PANI.

The crystallographic structures of GO, PANI, and the GO-PANI nanocomposite were investigated using XRD analysis, as shown in Fig. 4a and b, and 4c. The XRD pattern for GO (Fig. 4a) exhibits a sharp and intense peak around 2θ = 10°, corresponding to the (002) plane. This peak is characteristic of GO and indicates the successful oxidation of graphite^37^. In Fig. 4b, the XRD pattern for PANI displays peaks at 2θ = 15.2°, 20.12°, and 25.37°, which correspond to the PANI structure^38^.

Previous studies have recorded a broad diffraction peak for GO at 10°, signifying the successful conversion from graphite to graphene oxide. The observed reduction in the intensity and degree of crystallinity of GO, as seen in Fig. 4c, may be attributed to the intercalation of PANI chains between GO sheets, which results in increased spacing between the GO layers due to the incorporation of the polymer matrix^36^. Furthermore, the presence of PANI peaks in the XRD pattern of the GO-PANI nanocomposite suggests that the molecular structure of PANI remains intact and is not damaged during the reaction. Additionally, the absence of the characteristic GO peak in the XRD pattern indicates that GO did not undergo agglomeration and was nearly completely reacted during the polymerization process^39^.

**Fig. 5:**
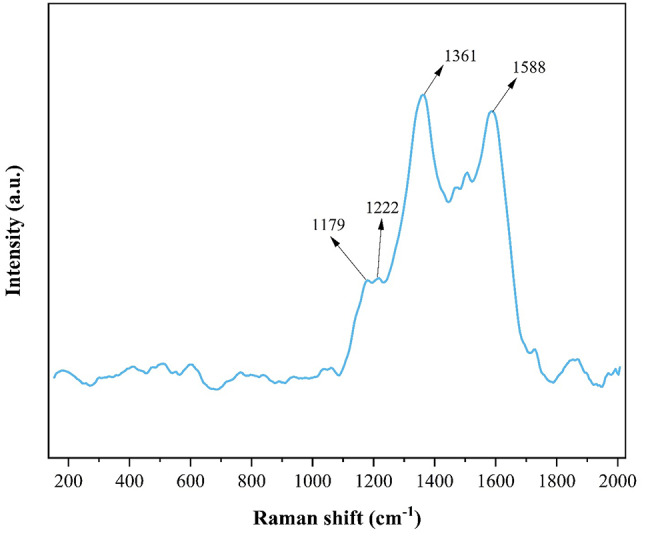
Raman spectrum of GO/PANI nanocomposite.

The Raman spectrum of the GO/PANI nanocomposite reveals several distinct peaks that correspond to various vibrational modes of the composite’s constituent materials, providing valuable insights into its structural and electronic properties (Fig. 5).

Moving to the medium-frequency range, the peaks at 1179 cm⁻¹ and 1222 cm⁻¹ are characteristic of C-H bending vibrations in the quinoid and benzenoid rings of PANI, respectively. These vibrations are key signatures of the conducting form of PANI, indicating that the polymer within the composite is in a doped, conductive state.

At higher frequencies, the prominent peaks at 1361 cm⁻¹ and 1588 cm⁻¹ correspond to the D and G bands of GO, respectively^40^. The D band at 1361 cm⁻¹ is associated with the presence of defects in the GO structure, such as those introduced by oxygen-containing functional groups during oxidation. The G band at 1588 cm⁻¹ reflects the graphitic nature of the material, representing the in-plane vibrations of sp² bonded carbon atoms. The presence of both the D and G bands confirms the successful incorporation of GO into the composite, with the D band indicating defect sites that can play a role in the material’s reactivity and functionality^41,42^.

**Fig. 6:**
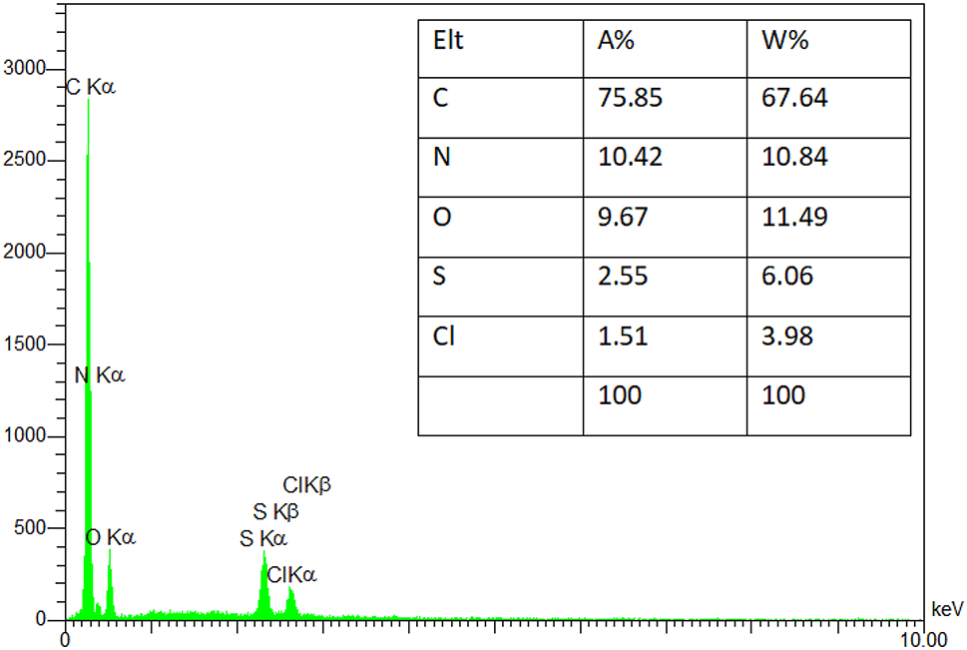
EDS pattern of GO-PANI nanocomposite.

EDS analysis confirmed the presence of various elements in the GO-PANI nanocomposite (Fig. 6), validating the success of the synthesis process. The detection of carbon and oxygen elements confirms the presence of GO, while the identification of nitrogen elements indicates the incorporation of PANI into the GO-PANI nanocomposite^39^. The presence of chlorine ions suggests the formation of ionic bonds with nitrogen in polyaniline. Despite repeated washes, the structure of the nanocomposite retains sulfur traces from ammonium persulfate and H_2_SO_4_, demonstrating the washing process may not have completely removed all by-products, highlighting the need for thorough cleaning during synthesis^33^. These findings offer valuable insights into the composition of the GO-PANI nanocomposite and can aid in further optimizing its properties for various applications.

**Fig. 7:**
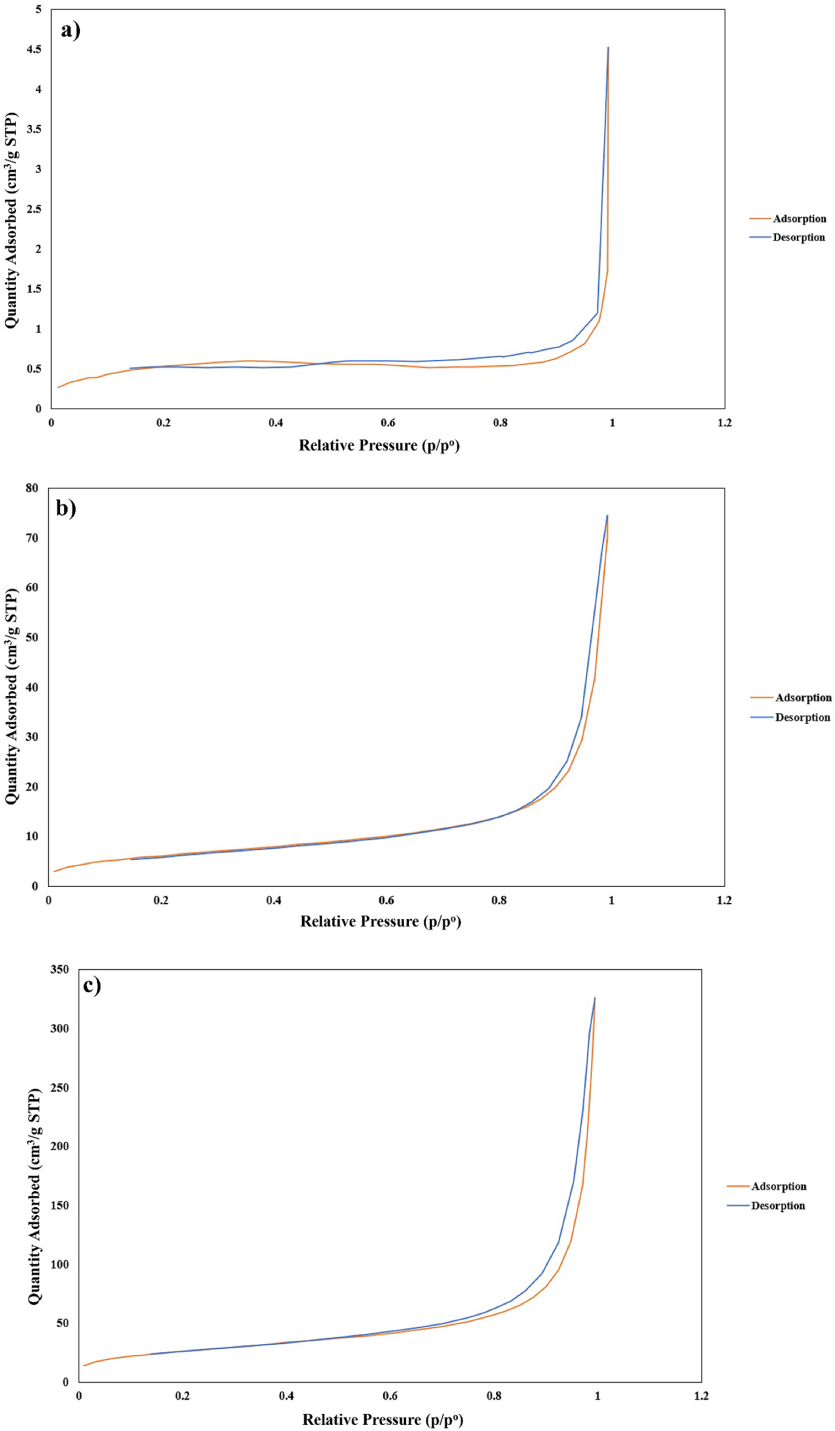
Isotherm type of (**a**) GO, (**b**) PANI, and (**c**) GO/PANI nanocomposite.

The BET analysis was employed to measure the surface area, pore size, and pore volume of the GO, PANI, and GO/PANI nanocomposite. The isotherm presented in Fig. 7, which illustrates the relationship between relative pressure and the quantity of gas adsorbed/desorbed, is characteristic of a Type IV isotherm. This type of isotherm is typically associated with mesoporous materials, characterized by pores in the range of 2 to 50 nm. The presence of a hysteresis loop, observed between the adsorption and desorption curves, confirms the mesoporous nature of the material. This loop is attributed to capillary condensation occurring within the mesopores, a phenomenon commonly observed in materials with such pore structures. The specific surface area of the GO/PANI composite, as determined by BET analysis, is 134.65 m²/g. This substantial surface area is advantageous as it enhances the material’s capacity to interact with other substances, thus improving its effectiveness in processes requiring extensive surface interactions. The average pore size of the GO/PANI composite is approximately 107.64 Å (10.76 nm), situating it firmly within the mesoporous category. The total pore volume of 0.5084 cm³/g further corroborates the mesoporous nature of the composite (Table 1). These properties indicate that the GO/PANI composite not only possesses a large surface area but also a significant internal volume for adsorption. This combination of features renders the material particularly suitable for applications where both surface area and pore structure are critical, such as catalysis, pollutant adsorption, and energy storage devices.

**Table 1:** The surface area, pore size, and pore volume of (a) GO, (b) PANI, and (c) GO/PANI nanocomposite.

Entry	Name	S_(BET)_$\:\left(\frac{{\mathbf{m}}^{2}}{\mathbf{g}}\right)$	Pore Size $\left( {\mathop {\text{A}}\limits^{{\text{o}}} } \right)$	Pore Volume$\:\left(\frac{{\mathbf{c}\mathbf{m}}^{3}}{\mathbf{g}}\right)$
a	GO	2.9667	485.829	0.006664
b	PANI	32.1460	199.596	0.116211
c	GO-PANI catalyst	134.6465	107.6405	0.508439

**Fig. 8:**
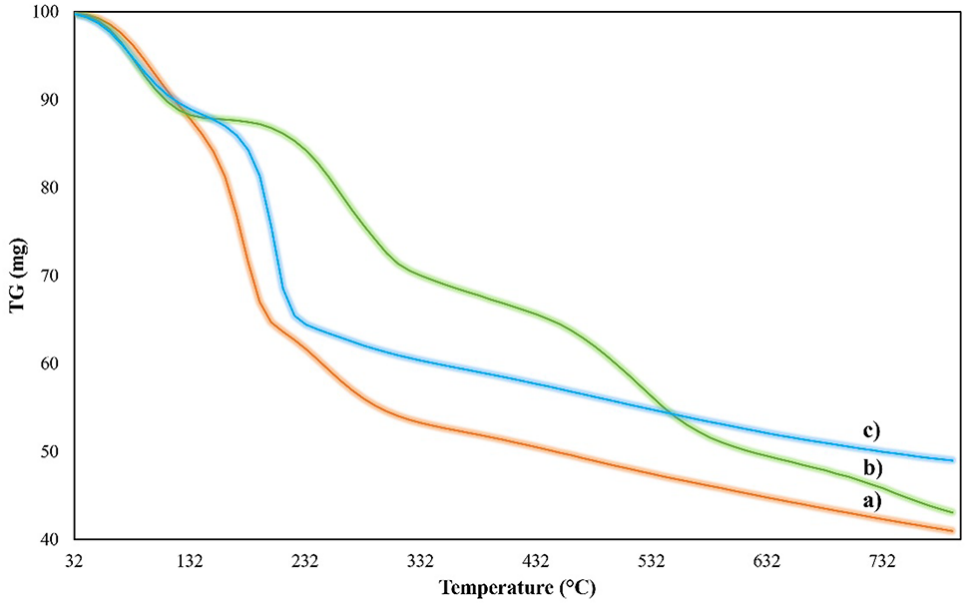
TGA analysis of (**a**) GO, (**b**) PANI, and (**c**) GO-PANI.

The thermal stability of GO, PANI, and GO-PANI nanocomposite was thoroughly analyzed and investigated through TGA analysis as depicted in Fig. 8.

The TGA curve for GO (Fig. 8-a) shows a significant weight loss occurring in two main stages. The first stage, around 100–150 °C, is likely due to the loss of absorbed water and other volatile components. Graphene oxide is known to contain a large number of oxygen-containing functional groups (hydroxyl, epoxy, and carboxyl groups) that can absorb moisture. The second major weight loss occurs around 200–300 °C, corresponding to the decomposition of the oxygen-containing functional groups and the loss of carbon dioxide, carbon monoxide, and water. This weight loss is typical for GO and is associated with the thermal reduction of GO, leading to the removal of oxygen functionalities and the conversion of GO to a more reduced form^39^.

The TGA curve for PANI (Fig. 8-b) demonstrates a more gradual weight loss over the temperature range. The first weight loss, occurring below 100 °C, is attributed to the removal of moisture and any residual solvents. The subsequent weight loss between 200 and 400 °C corresponds to the thermal degradation of the PANI backbone. This degradation is associated with the breakdown of the polymer chains, including the loss of dopants, hydrogen, and nitrogen-containing groups^43,44^. PANI typically has better thermal stability compared to GO, which is reflected in the higher decomposition temperature and more gradual weight loss.

The TGA curve for the GO/PANI composite (Fig. 8-c) shows an intermediate thermal behavior between GO and PANI. The initial weight loss, similar to GO and PANI, occurs below 150 °C due to the loss of moisture and volatiles. The subsequent weight loss between 200 and 400 °C is indicative of the decomposition of both GO and PANI components. However, the composite exhibits enhanced thermal stability compared to pure GO, as indicated by the delayed and reduced weight loss at higher temperatures. This improved thermal stability can be attributed to the interaction between GO and PANI, where the PANI may act as a protective barrier, reducing the rate of decomposition of the GO component^45–47^.

### The catalytic activity of GO-PANI nanocomposite

#### Optimization of reaction conditions for synthesizing 1,4-dihydropyridine and hydroquinoline derivatives

**Fig. 9:**
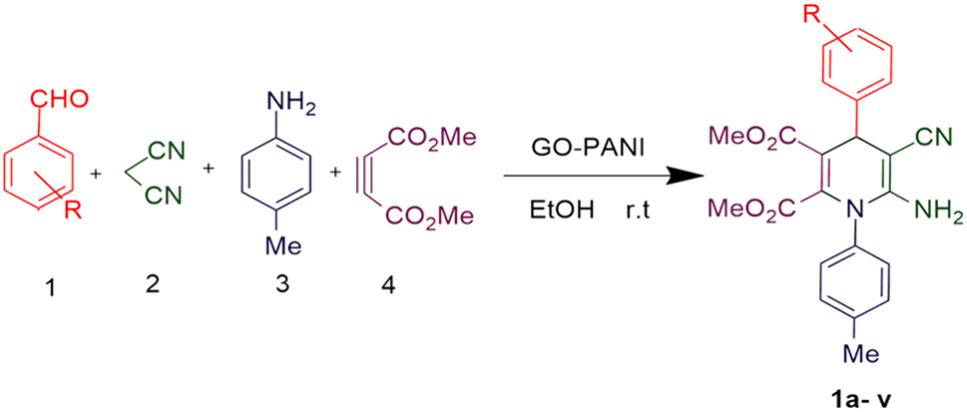
Synthesis of 1,4-dihydropyridine derivatives.

**Fig. 10:**
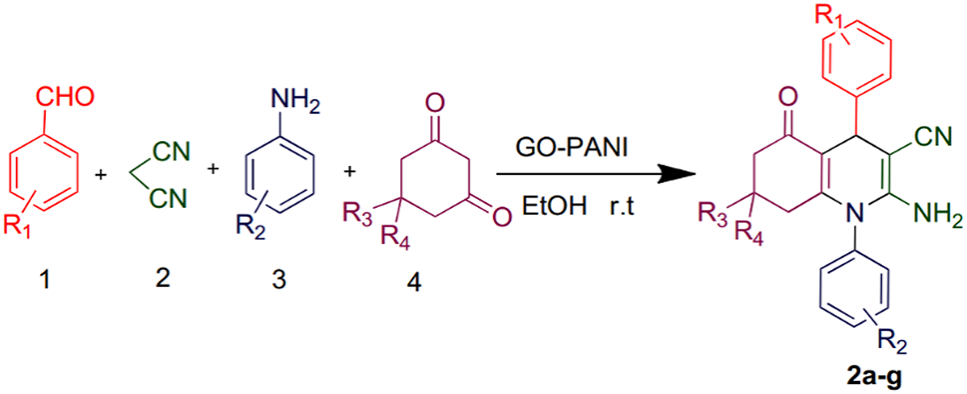
Synthesis of hydroquinoline derivatives.

To determine the optimal reaction conditions, two model reactions were selected. Model reaction A, aimed at synthesizing 1,4-dihydropyridine derivatives, involved the reaction of 4-nitrobenzaldehyde (1 mmol), malononitrile (1 mmol), DMAD (1 mmol), and aniline (1 mmol). Model reaction B, focused on synthesizing hydroquinoline derivatives, involved the reaction of 3-nitrobenzaldehyde (1 mmol), malononitrile (1 mmol), dimedone (1 mmol), and methylaniline (1 mmol) (as shown in Figs. 9 and 10). The desired product yields were obtained through crystallization. Various parameters, including temperature, solvent type, catalyst amount, and reaction time, were systematically investigated to optimize the reaction conditions.

**Fig. 11:**
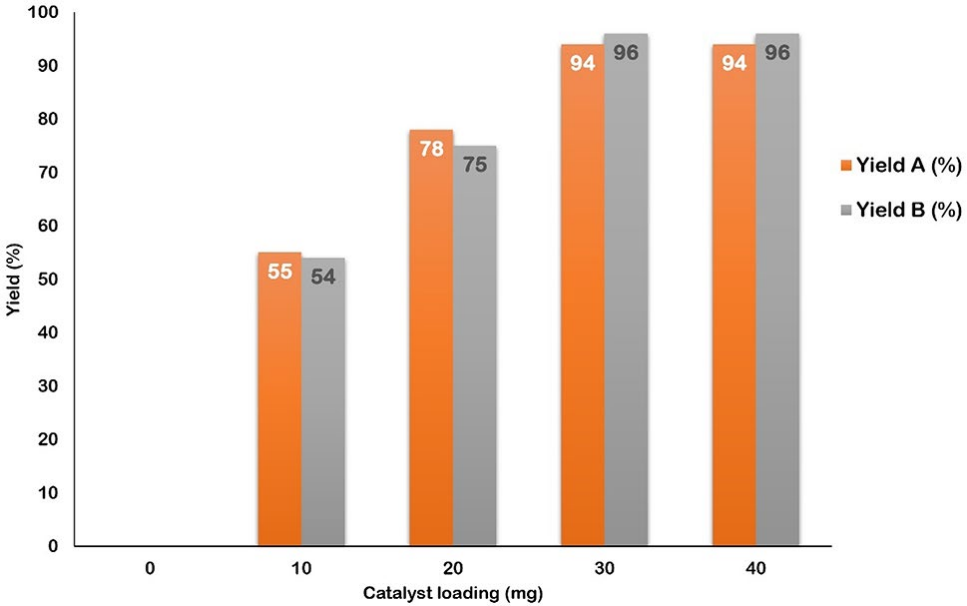
Effect of catalyst loading on yield of model reactions. Model reaction (**A**): 4-nitrobenzaldehyde (1 mmol), malononitrile (1 mmol), DMAD (1 mmol), and aniline (1 mmol). Model reaction (**B**): 3-nitrobenzaldehyde (1 mmol), malononitrile (1 mmol), dimedone (1 mmol) and methylaniline (1 mmol).

In the initial step, the model reactions were conducted without a catalyst at room temperature, with progress monitored by thin-layer chromatography (TLC). The results indicated that no desired product was formed, underscoring the necessity of a catalyst for the reaction to proceed. Subsequently, varying amounts of the GO-PANI nanocomposite (10, 20, 30, and 40 mg) were tested to optimize the reaction efficiency. The findings revealed that increasing the catalyst amount beyond 30 mg did not enhance the reaction efficiency. Consequently, the optimal reaction efficiency was achieved with 30 mg of the GO-PANI catalyst (Fig. 11).

The yields related to the isolated product.

**Fig. 12:**
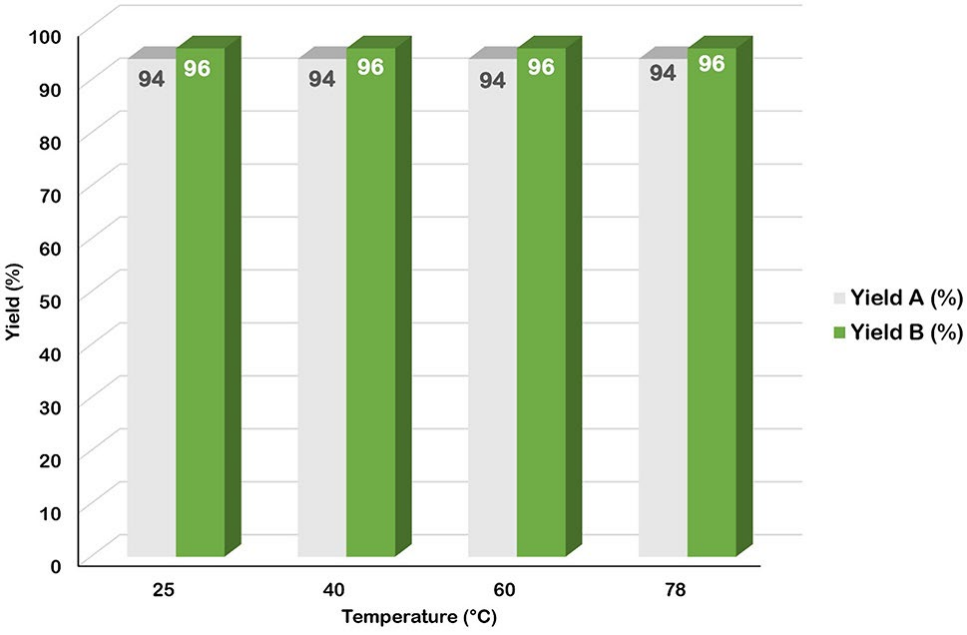
Effect of reaction temperature on yield of model reactions. Model reaction (**A**): 4-nitrobenzaldehyde (1 mmol), malononitrile (1 mmol), DMAD (1 mmol), and aniline (1 mmol). Model reaction (**B**): 3-nitrobenzaldehyde (1 mmol), malononitrile (1 mmol), dimedone (1 mmol) and methylaniline (1 mmol).

Subsequently, the effect of temperature on the performance of the GO-PANI catalyst (30 mg) was investigated at 25 °C, 40 °C, 60 °C, and reflux temperature. The experimental results indicated that the reaction efficiency remained consistent across the different temperatures tested (Fig. 12). Consequently, room temperature was selected as the optimal temperature to minimize energy consumption and reduce the overall cost of the reaction. This choice allows the reaction to be conducted more economically while maintaining consistent efficiency.

The yields related to the isolated product.

**Fig. 13:**
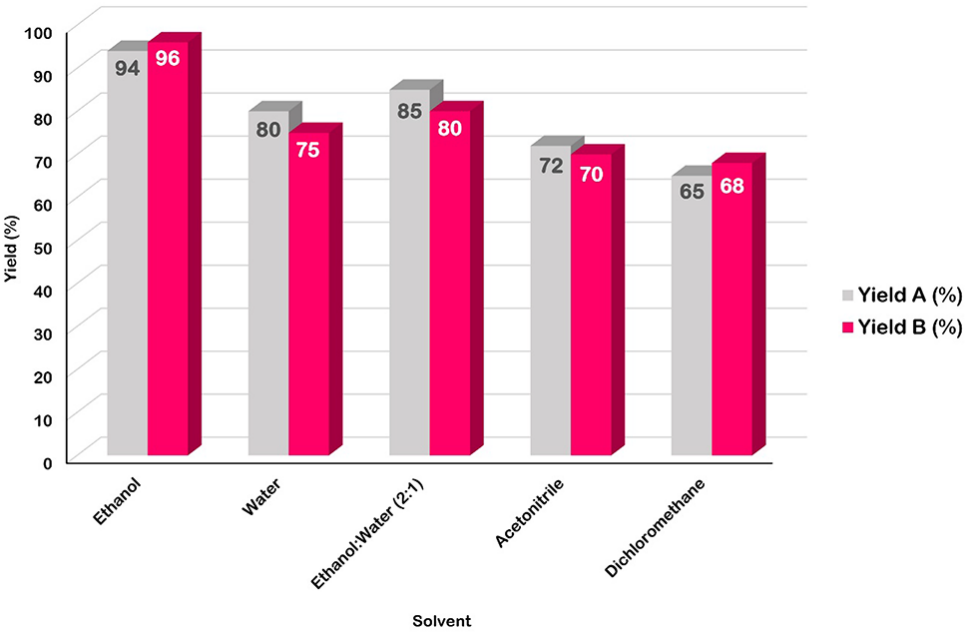
Effect of solvent on yield of model reactions. Model reaction (**A**): 4-nitrobenzaldehyde (1 mmol), malononitrile (1 mmol), DMAD (1 mmol), and aniline (1 mmol). Model reaction (**B**): 3-nitrobenzaldehyde (1 mmol), malononitrile (1 mmol), dimedone (1 mmol) and methylaniline (1 mmol).

Subsequently, the effects of polar and non-polar solvents on the model reactions were investigated to determine the optimal solvent. Water and ethanol were selected as polar solvents, while acetonitrile and dichloromethane were used as non-polar solvents (Fig. 13). The results demonstrated that the reactions performed in polar solvents, particularly water and ethanol, exhibited higher efficiency compared to those in non-polar solvents. Ultimately, ethanol, a green solvent, was found to provide the best reaction efficiency. The superior performance of ethanol over water can be attributed to the fact that water forms more hydrogen bonds, leading to the production of more intermediate products. This increased formation of intermediates in water reduces the efficiency of the desired reaction.

The yields related to the isolated product.

**Fig. 14:**
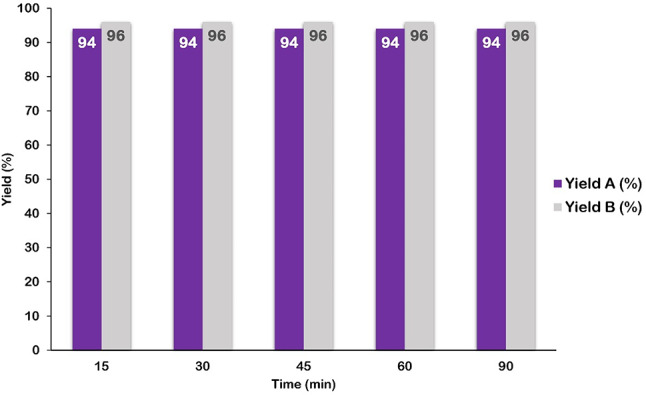
Effect of reaction time on yield of model reactions. Model reaction (**A**): 4-nitrobenzaldehyde (1 mmol), malononitrile (1 mmol), DMAD (1 mmol), and aniline (1 mmol). Model reaction (**B**): 3-nitrobenzaldehyde (1 mmol), malononitrile (1 mmol), dimedone (1 mmol) and methylaniline (1 mmol).

To optimize the reaction time, the model reaction was conducted at room temperature using ethanol as the solvent and 30 mg of GO-PANI as the catalyst. The reaction efficiency was evaluated at intervals of 15, 30, 45, 60, and 90 min (Fig. 14) to determine the optimal duration. The results indicated that the reaction efficiency did not improve beyond 15 min; therefore, 15 min was identified as the optimal reaction time.

The yields related to the isolated product.

Model reactions were conducted using 30 mg each of GO, PANI, and the GO-PANI nanocomposite as catalysts, with ethanol as the solvent, at 25 °C for 15 min (Table 2). The reaction efficiency was analyzed in the presence of each component. The use of GO as a catalyst resulted in negligible reaction efficiency, which can be attributed to the limited number of functional groups in GO, restricting its participation in the reaction. Additionally, GO’s bulky structure hinders the formation of hydrogen bonds, which are essential for the reaction to proceed.

In contrast, PANI, which contains a large number of amino groups, is capable of forming many hydrogen bonds. However, due to its inadequate surface area and low stability, the reaction yield was still relatively low. Moreover, the challenges associated with recycling and reusing PANI can lead to increased operational costs and waste generation, negatively impacting the overall sustainability and economic viability of the catalytic process.

When PANI composited with GO, the number of active sites increased, leading to the highest level of reaction efficiency observed with the GO-PANI nanocomposite. The synergy between the two components and increased surface area facilitated the formation of more hydrogen bonds, thereby enhancing the reaction. The GO-PANI nanocomposite provided a significantly higher number of active sites compared to GO or PANI alone, resulting in superior reaction efficiency.

**Table 2:** Comparison of GO-PANI with other Catalytic conditions.

Entry	Catalyst	Solvent	Model reaction A yield^a, c^ (%)	Model reaction B yeld^b, c^ (%)
1	GO	ETOH	Trace	Trace
2	PANI	ETOH	48	52
3	GO-PANI	ETOH	94	96

^a^Reaction conditions: 4-nitrobenzaldehyde (1 mmol), malononitrile (1 mmol), DMAD (1 mmol), and methylaniline (1 mmol), at room temperature for 15 min.

^b^Reaction conditions: 3-nitrobenzaldehyde (1 mmol), malononitrile (1 mmol), dimedone (1 mmol) and methylaniline (1 mmol), at room temperature for 15 min.

^c^The yields related to the isolated product.

For indicating the catalytic merits of GO-PANI nanocomposite, the model reactions were applied by the different catalysts and shown in Table 3. A small amount of product can be seen by using LiOH, Ln_2_O_3_, and PPh_3_ catalysts and spending a long reaction time (Table 3, entries 2, 3, and 5). However, the reaction efficiency reaches higher in the presence of K_2_CO_3_, KOH, Na_2_CO_3_, and NaHCO_3_ catalysts. However, the long reaction time required in the presence of the mentioned catalysts makes these catalysts undesirable (Table 3, entries 1,4,6,7).

According to Table 3, the most favorable reaction efficiency was obtained in the presence of GO-PANI nanocomposite (Table 3, entry 8). Based on the experimental results, it was found that the GO-PANI nanocomposite demonstrates greater efficiency with shorter reaction times and improved performance when compared to previously reported nanocomposites. The synthesized GO-PANI nanocomposite was also found to act as a highly effective heterogeneous catalyst, which can be easily separated through a simple filtration process^48–51^.

In our study, we wanted to assess the effectiveness of the model reactions by conducting a comprehensive analysis of the reaction conditions and efficiency. We compared the results with other published literature to understand better the performance of the catalysts used in similar studies. The data presented in Tables 4 and 5 revealed that the use of GO-PANI nanocomposite in model reactions outperformed other catalysts in terms of efficiency and reaction conditions.

In addition to superior performance, the GO-PANI nanocomposite we used as a non-metal catalyst offered several other advantages over other published literature. Firstly, the reaction time was significantly reduced, which is a crucial factor when working with time-sensitive reactions. Secondly, the catalyst was easy to separate, allowing for quick and efficient recovery. Thirdly, the efficiency of the reaction was exceptionally high, demonstrating the potential of GO-PANI nanocomposite as a promising candidate for future studies. Furthermore, the work-up process was straightforward and hassle-free, simplifying the overall procedure. Finally, the ability to reuse the catalyst multiple times made it a cost-effective and sustainable option.

On the other hand, with the optimized reaction conditions, various aldehyde and amine derivatives (electron-donating and electron-withdrawing groups) were utilized to investigate the catalytic activity of the synthesized nanocomposite (Tables 6 and 7).

**Table 3:** Comparison of GO-PANI with other time and Catalytic conditions.

Entry		Catalyst		Solvent	Time (h)	Model reaction A Yield^a, c^ (%)	Model reaction B Yield^b, c^ (%)	
1		K_2_CO_3_		EtOH	12	46	53	
2		LiOH		EtOH	16	Trace	Trace	
3		Ln_2_O_3_		EtOH	16	Trace	Trace	
4		KOH		EtOH	8	54	66	
5		PPh_3_		EtOH	16	Trace	Trace	
6		Na_2_CO_3_		EtOH	8	62	68	
7		NaHCO_3_		EtOH	8	66	70	
8		GO-PANI		EtOH	0.25	94	96	

^a^Reaction conditions: 4-nitrobenzaldehyde (1 mmol), malononitrile (1 mmol), DMAD (1 mmol), and methylaniline (1 mmol), at room temperature for 15 min.

^b^Reaction conditions: 3-nitrobenzaldehyde (1 mmol), malononitrile (1 mmol), dimedone (1 mmol) and methylaniline (1 mmol), at room temperature for 15 min.

^c^The yields related to the isolated product.

**Table 4:** Comparison of model reaction A with other time and Catalytic Conditions^a^.

Entry	Catalyst	Reaction condition	Time (Min)	Yield (%)	References
1	[3,6-DOMDA]OTf	CH_2_Cl_2_, reflux	30	87	^52^
2	NaOH	EtOH, r.t	10	78	^50^
3	Rose bengal	H_2_O: EtOH (9:1) blue LED	40	95	^53^
4	GO-PANI nanocomposite	EtOH, r.t	15	94	This work

^a^Reaction conditions: 4-nitrobenzaldehyde (1 mmol), malononitrile (1 mmol), DMAD (1 mmol), and methylaniline (1 mmol), at room temperature for 15 min.

**Table 5:** Comparison of model reaction B with other time and Catalytic Conditions^a^.

Entry	Catalyst	Reaction Condition	Time (Min)	Yield (%)	References
1	DBU	EtOH, 80 ^o^C Microwave	5	92	^54^
2	DABCO/TEAB	Water, reflux	15	88	^55^
3	MnFe_2_O_4_@SiO_2_–Pr– NH@BTA@Cu(OAc)_2_	EtOH, r.t	20	83	^30^
4	GO-PANI nanocomposite	EtOH, r.t	15	96	This work

^a^Reaction conditions: 3-nitrobenzaldehyde (1 mmol), malononitrile (1 mmol), dimedone (1 mmol) and methylaniline (1 mmol), at room temperature for 15 min.

**Table 6:** Reaction of aldehyde, malononitrile, DMAD, and aniline derivatives for synthesizing 1,4-dihydropyridine in optimized reaction conditions.


Entry		R_1_			R_2_		R_3_		Product	Time (mine)		Yield^a, b^ (%)		M.P (^o^C)		Report		
														Found				
1a		3-NO_2_			Me		Me			15		94		210–212		210-212^48^		
2a		3-NO_2_			4-Cl		Me			15		92		194–195		193-195^49^		
3a		4-Br			Me		Me			15		90		186–188		185-187^50^		
4a		4-Cl			Me		Me			15		88		187–188		186-188^50^		
5a		H			Me		Me			15		82		164–166		165-167^48^		
6a		4-OMe			Me		Me			20		86		181–183		182-183^51^		
7a		3-NO_2_			4-OMe		Me			15		93		186–187		185-187^48^		
8a		4-Cl			4-OMe		Me			15		90		184–186		185-187^51^		
9a		4-OMe			4-OMe		Me			20		83		158–160		159-161^50^		
10a		4-Cl			4-Cl		Me			15		92		129–131		130-132^48^		
11a		4-Cl			4-Br		Me			15		92		182–183		181-183^51^		
12a		H			H		Me			15		80		160–162		161-163^56^		
13a		3-NO_2_			H		Me			15		90		216–219		217-219^49^		
14a		4-NO_2_			4-Me		Et			15		92		168–171		170-172^57^		
15a		4-OMe			4-Br		Et			20		89		173–175		175-177^57^		
16a		H			H		Et			15		83		159–160		160-162^57^		

**Table 7:** Reaction of aldehyde, malononitrile, dimedone, and aniline derivatives for the synthesis of hydroquinoline in optimized reaction conditions.


Entry			R_**1**_	**R**_**2**_		**R**_**3**_		Product			Time (mine)		Yield^**a, b**^ (%)		M.P (^o^C )		Report
															Found		
1b			4-Cl	H		H					30		90		227–229		228-230^58^
2b			H	H		H					34		87		150–151		149-151^59^
3b			4-Me	H		H					40		84		234 (dec)		235 (dec)^58^
4b			4-Me	4-Me		Me					40		94		252–254		252-254^60^
5b			3-NO_2_	4-Me		Me					30		96		275–277		277-279^31^
6b			3-NO_2_	H		Me					30		93		269–270		268-269^60^
7b			3-NO_2_	4-Br		Me					35		91		271–273		271-272^61^

aReaction conditions: 4-nitrobenzaldehyde (1 mmol), malononitrile (1 mmol), dimedone (1 mmol) and methylaniline (1 mmol), at room temperature for 15 min.

bThe yields related to the isolated product.

### Mechanism of synthesizing 1,4-dihydropyridine derivatives

**Fig. 15:**
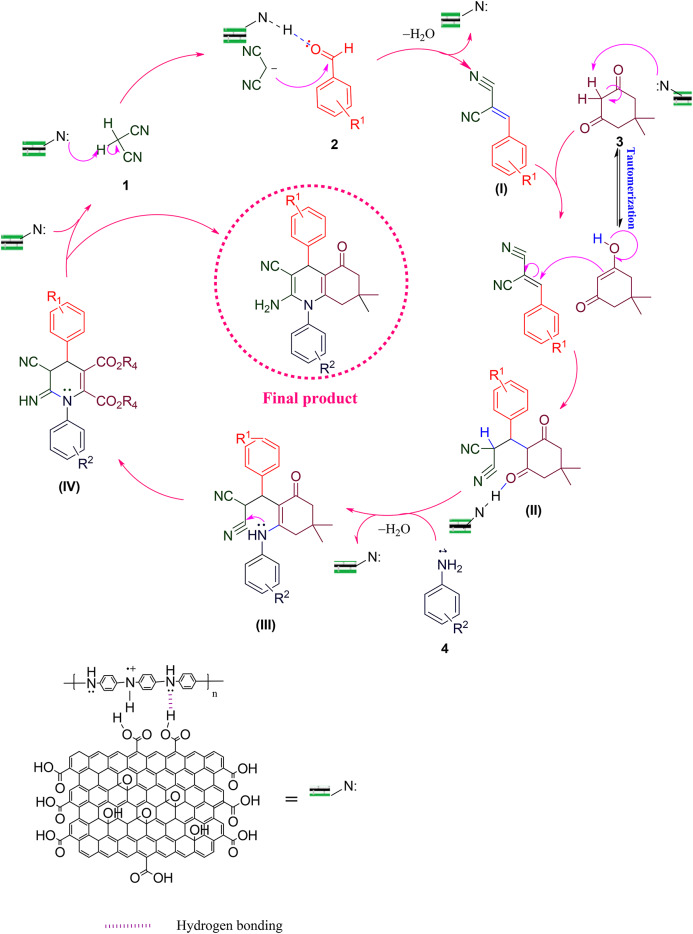
Mechanism of N-aryl-1,4-dihydropyridine derivatives synthesis.

**Fig. 16:**
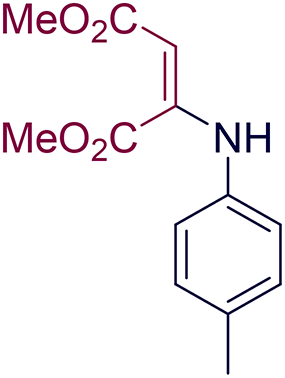
Intermediate (III) of the mechanism of synthesized 1,4-dihydropyridine derivatives.

According to the results, a reasonable mechanism for synthesizing 1,4-dihydropyridine by GO-PANI is shown in Fig. 15^22^. The catalyst active sites to create a hydrogen bond are the pair of nitrogen electrons of polyaniline. This causes the reaction to form a hydrogen bond or as nucleophile in each mechanism step. Initially, malononitrile (1) and aldehyde (2) undergo Knoevenagel condensation, wherein the nitrogen electron pairs in PANI interact with the acidic hydrogen in malononitrile, leading to its separation. Subsequently, malononitrile acts as a nucleophile, reacting with the activated carbonyl in aldehyde as the oxygen electron pairs interact with PANI. Then, the removal of water results in the formation of intermediate (I). Next, zwitterrionate Intermediate (II) is formed when aniline (3) reacts with β-ketoester (4) in the second step. In the next step, the Michael addition occurs between intermediates (I) and (II) by forming a 6-membered ring, leading to the formation of intermediate (III). Finally, the intramolecular cyclization in the structure leads to the synthesis of 1,4 dihydropyridine derivatives (5)^62,63^. Moreover, the intermediate (III) (Fig. 16) was synthesized (dimethyl p-tolylaminofumarate (MP: 72–74 °C^64^), and the reaction was initiated from it to prove this mechanism. Also, its FT-IR spectra can be seen in Supporting information file.

### Mechanism of synthesizing hydroquinoline derivatives

**Fig. 17:**
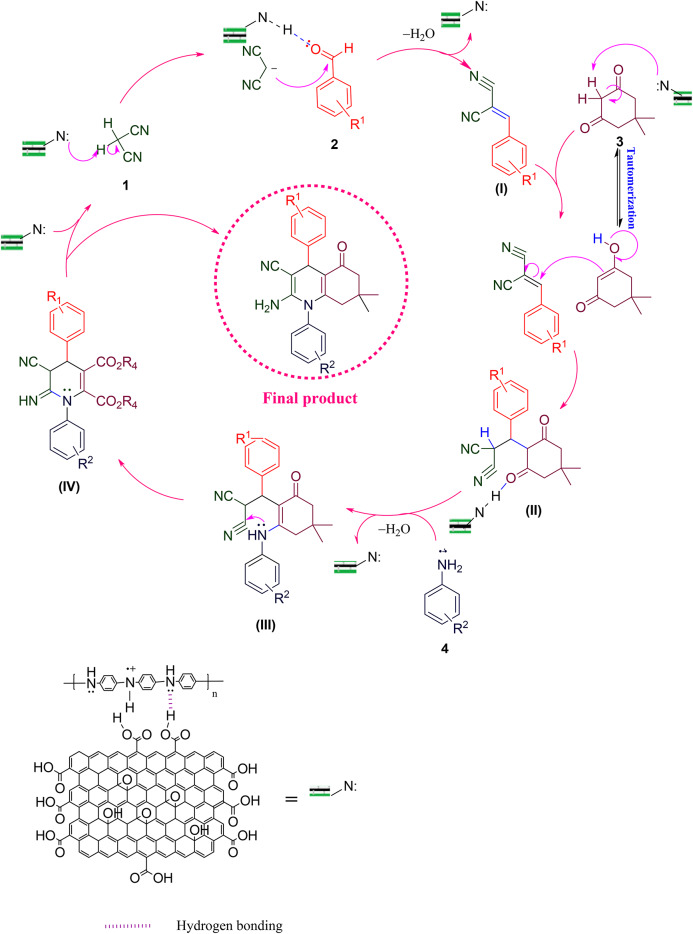
Mechanism of hydroquinoline derivatives synthesis.

**Fig. 18:**
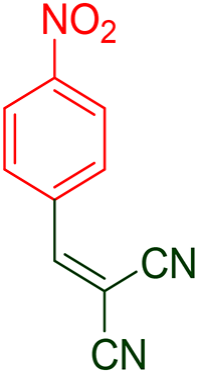
Intermediate (II) of the mechanism of synthesized hydroquinoline derivatives.

According to the obtained results, a plausible mechanism for the synthesis of hydroquinoline by the GO/PANI catalyst is shown in Fig. 17. In the first step, malononitrile (1) and aldehyde (2) undergo Knoevenagel condensation, where the nitrogen electron pairs in PANI interact with the acidic hydrogen in malononitrile, causing it to separate. Then, malononitrile acts as a nucleophile and reacts with the activated carbonyl in aldehyde, with the oxygen electron pairs interacting with PANI. The subsequent removal of water leads to the formation of intermediate (I). Dimedone (3) possesses an acidic hydrogen that reacts with the nitrogen pair of PANI, leading to separation. Subsequently, the dimedone acts as a nucleophile, undergoing a Michael addition reaction with intermediate I to form intermediate (II). In intermediate II, the catalyst activates carbonyl dimedone, enabling aniline (4) to interact with the carbonyl carbon. After the water is removed, compound III is obtained. At this stage, the desired product, hydroquinoline (5), is obtained through intramolecular cyclization and intra-ring tautomerization^62,63^. Moreover, the intermediate (II) (Fig. 18) was synthesized (4-Nitrobenzylidenemalononitrile (MP: 153–155 °C^65^), and the reaction was initiated from it to prove this mechanism. Also, its FT-IR spectra can be seen in Supporting information file.

### Reusability of GO-PANI catalyst

**Fig. 19:**
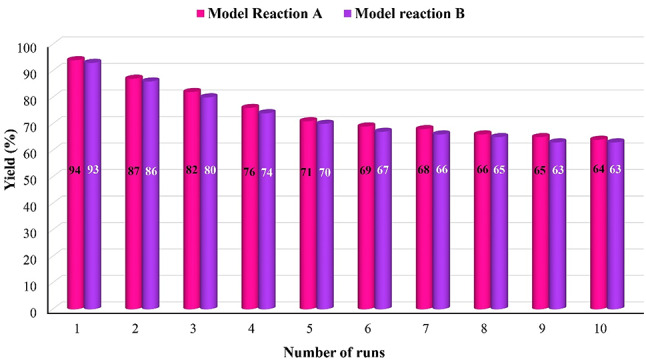
Reusability of GO-PANI catalyst.

**Fig. 20:**
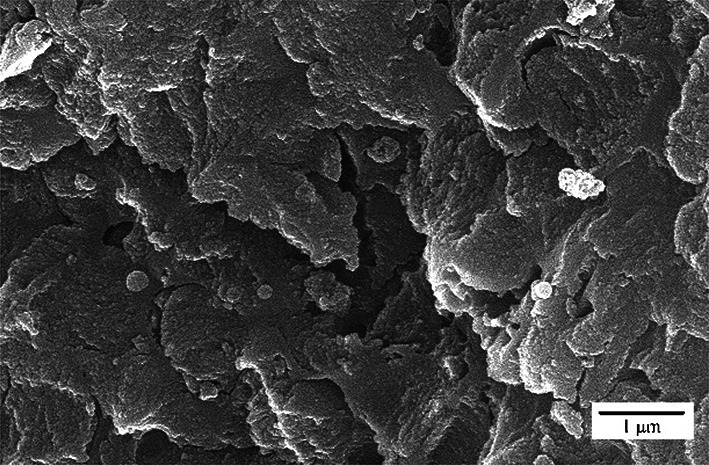
The FE-SEM image of recycled GO/PANI.

**Fig. 21:**
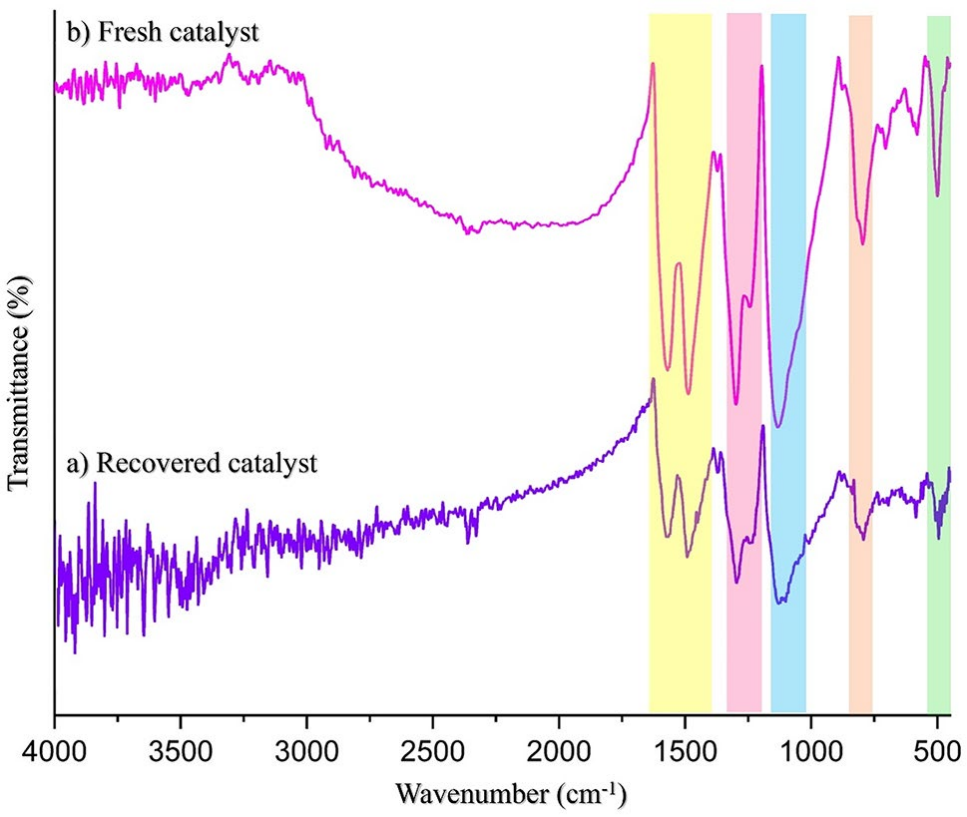
The FT-IR analysis of recycled GO/PANI.

Heterogeneous catalyst recovery plays a pivotal role in promoting the principles of green chemistry in organic reactions by enabling the efficient reuse of catalysts, minimizing waste, reducing environmental impact, and promoting sustainable processes. An essential aspect of this research involves investigating the catalyst’s reusability in synthesizing 1,4-dihydropyridine and hydroquinoline derivatives by exploring the catalyst recycling process. To test the catalyst’s reusability (0.1 g), the experiment was performed ten times, using optimal conditions in model reactions. After each trial, the catalyst was carefully separated through filtration, washed with ethanol, and then dried at 70 °C. The experiment results indicate that the catalyst efficiency remains remarkable even after undergoing ten recovery rounds(0.063 g), as shown in Fig. 19. This suggests that the catalyst’s reusability is a promising avenue for promoting eco-friendly and green chemistry practices. Moreover, the FE-SEM image and FT-IR analysis of recycled GO/PANI has been taken and is shown in Figs. 20 and 21, respectively. The synthesis can be easily scaled by proportionally increasing the quantities of starting materials while keeping the reaction conditions constant. Preliminary scaling experiments up to 10 times the original batch size were conducted without any significant change in the nanocomposite’s structural or catalytic properties.

### Future potential applications

The GO-PANI nanocomposite can be extended to other multi-component reactions (MCRs) due to its ability to bring multiple reactants together efficiently. This includes the synthesis of various heterocyclic compounds, such as pyrimidines, quinolines, and imidazoles, which are of significant interest in pharmaceutical and agrochemical industries. Also, The GO-PANI nanocomposite has the potential to be used in the degradation of organic pollutants in wastewater. Its catalytic properties can facilitate the breakdown of dyes, pesticides, and other harmful substances, contributing to environmental remediation efforts. The presence of functional groups in GO can allow the nanocomposite to adsorb heavy metals from contaminated water, making it a valuable material for water purification applications. Moreover, The composite can also function as a photocatalyst for the degradation of organic compounds under light irradiation. This application is particularly relevant for environmental cleanup efforts and sustainable chemical processes.

## Conclusion

The GO-PANI nanocomposite, a highly effective non-metal catalyst for the synthesis of heterocyclic compounds, was successfully synthesized through in-situ polymerization of aniline on GO nanosheets. During this process, the presence of GO facilitated the growth of PANI, as confirmed by FESEM and TEM studies, which revealed that the GO sheets served as templates for the polymerization. The synthesized nanocomposite demonstrated the ability to produce heterocyclic compounds such as 1,4-dihydropyridine and hydroquinoline derivatives with medicinal properties using a simple method, resulting in high yields. Furthermore, the GO-PANI nanocomposite maintained significant catalytic activity even after ten recovery cycles. The optimal conditions for synthesizing 1,4-dihydropyridine and hydroquinoline derivatives, were found to be at room temperature, using ethanol as a green solvent, with a reaction time of 15 min and a catalyst loading of 30 mg. The use of ethanol solvent as a green solvent, coupled with the absence of high temperature or energy requirements, makes this catalyst particularly advantageous for the synthesis of medicinal derivatives. Based on the findings of this research, the GO-PANI nanocomposite holds considerable promise for future applications in the synthesis of pharmaceutical compounds.

## Electronic supplementary material

Below is the link to the electronic supplementary material.

Supplementary Material 1

## Data Availability

The datasets used and/or analysed during the current study available from the corresponding author on reasonable request.
