# Supplementary material for: Catalytic efficiency of GO-PANI nanocomposite in the synthesis of N-Aryl-1,4-Dihydropyridine and hydroquinoline derivatives
Source: Sci Rep. 2025 Jan 22;15:2791. doi: 10.1038/s41598-024-82907-5 (PMC11754839; doi:10.1038/s41598-024-82907-5)
Supplement: Supplementary file 1 — Supplementary Material 1 [file 41598_2024_82907_MOESM1_ESM.docx]

*Supporting Information*

Catalytic Efficiency of GO-PANI Nanocomposite in the Synthesis of N-Aryl-1,4-Dihydropyridine and Hydroquinoline Derivatives

Hossein Ghafuri,*^a^ Moghadaseh keshvari ^a^, Fatemeh Eshrati ^a^, Peyman Hanifehnejad ^a^, Atefeh Emami ^a^, Hamid Reza Esmaili Zand ^a^

^a^ Catalysts and Organic Synthesis Research Laboratory, Department of Chemistry, Iran University of Science and Technology, 16846-13114 Tehran, Iran

*E-mail: ghafuri@iust.ac.ir

| **Contents** | **Page** |
| --- | --- |
| Title page | S1 |
| Chemical characterization of intermediates | S2 |
| Chemical characterization of 1,4-dihydropyridine derivatives | S3 |
| Chemical characterization of hydroquinoline derivatives | S6 |

**Chemical characterization of intermediates**

**Figure S1. FT-IR spectra of intermediate (III) of the mechanism of synthesized 1,4-dihydropyridine derivatives.**

**Figure S2. FT-IR spectra of intermediate (II) of the mechanism of synthesized hydroquinoline derivatives.**

**Chemical characterization of 1,4-dihydropyridine derivatives**

**Figure S3.** **FT-IR spectra of 1,4-dihydropyridine derivative.**


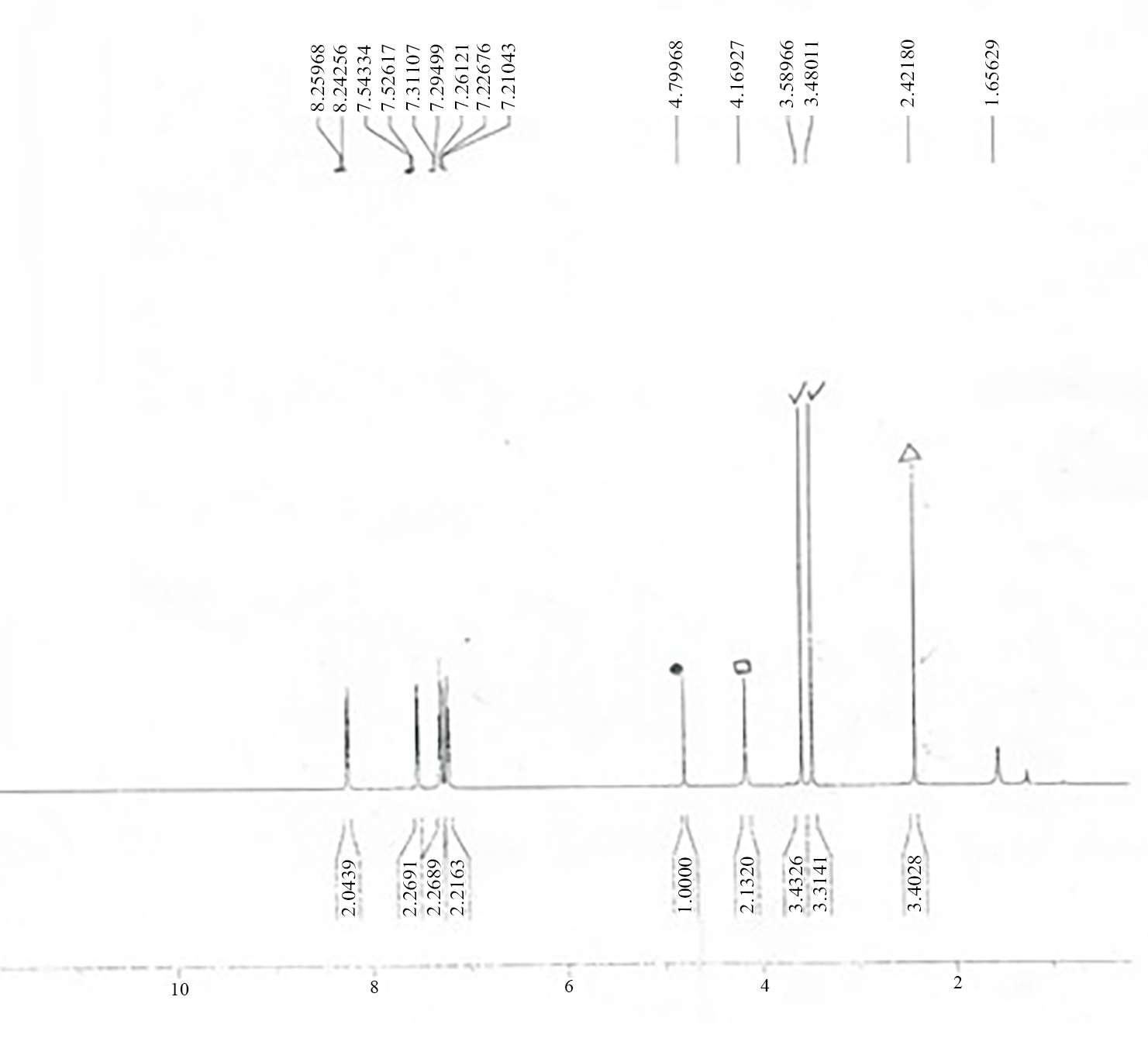


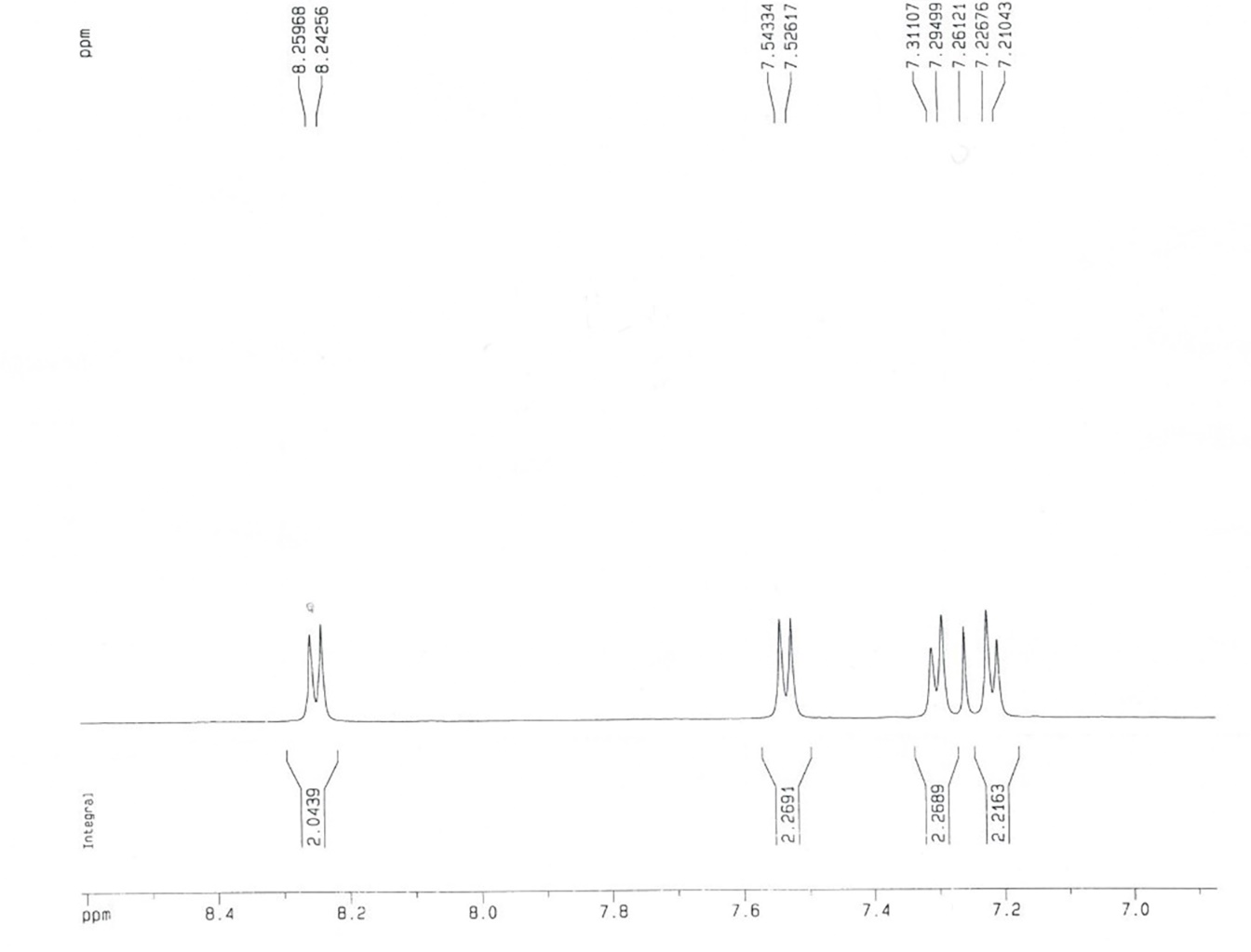


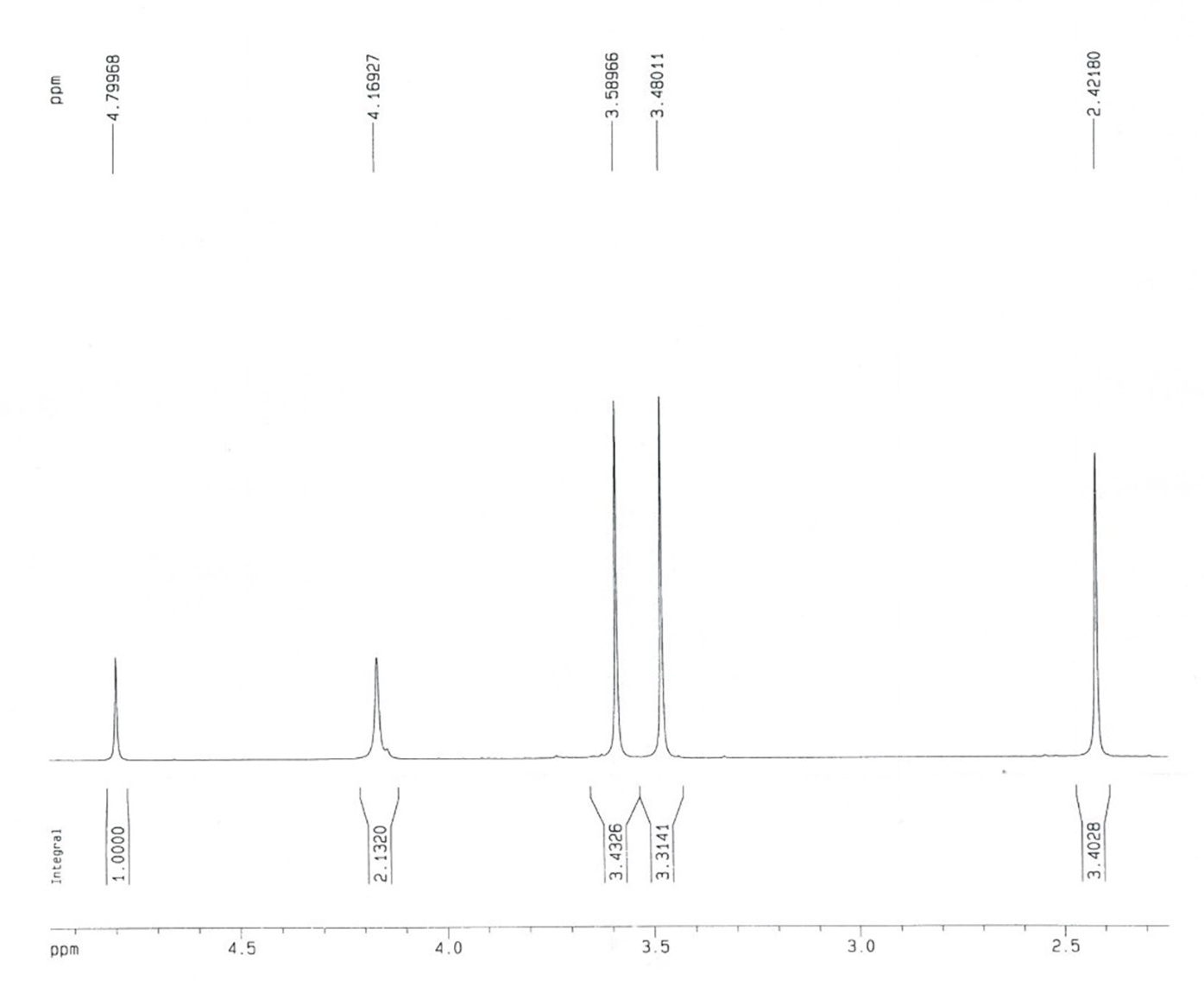


**Figure S4.** **^1^H NMR spectra of 1,4-dihydropyridine.**


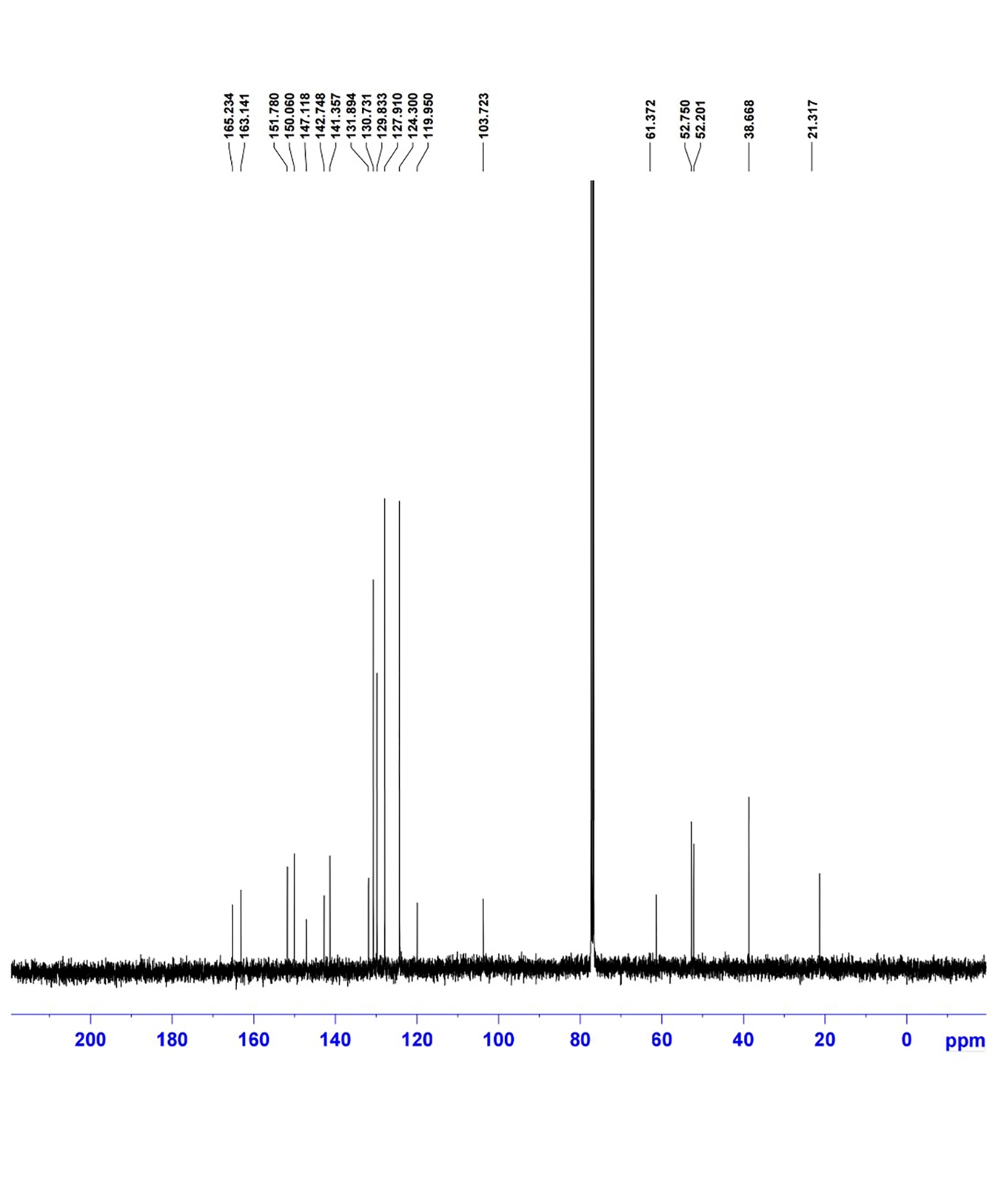
 **Figure S5. ^13^C NMR spectra of 1,4-dihydropyridine.**

**Chemical characterization of hydroquinoline derivatives**

**Figure S6. IR spectra of hydroquinoline.**


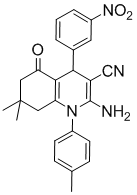

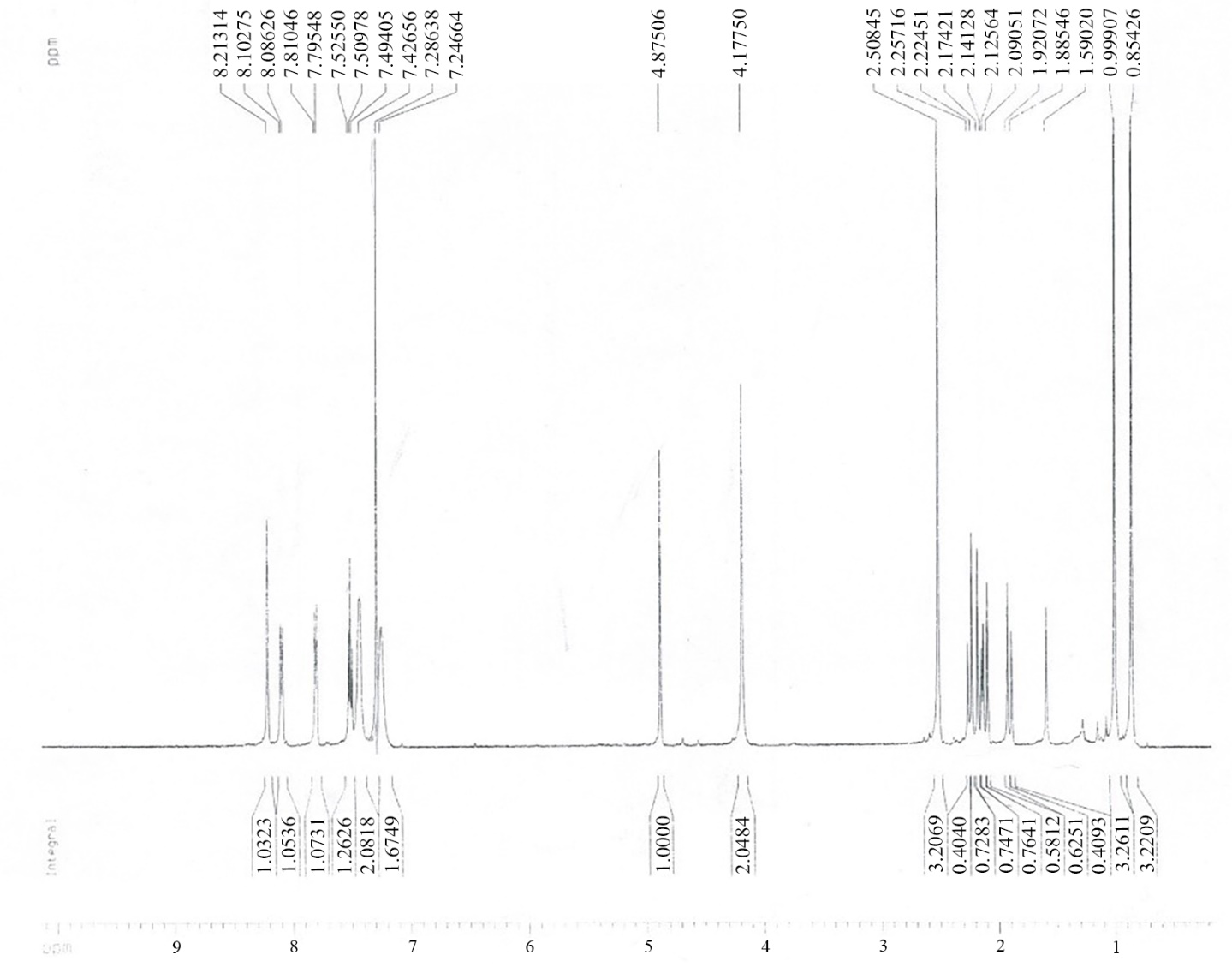


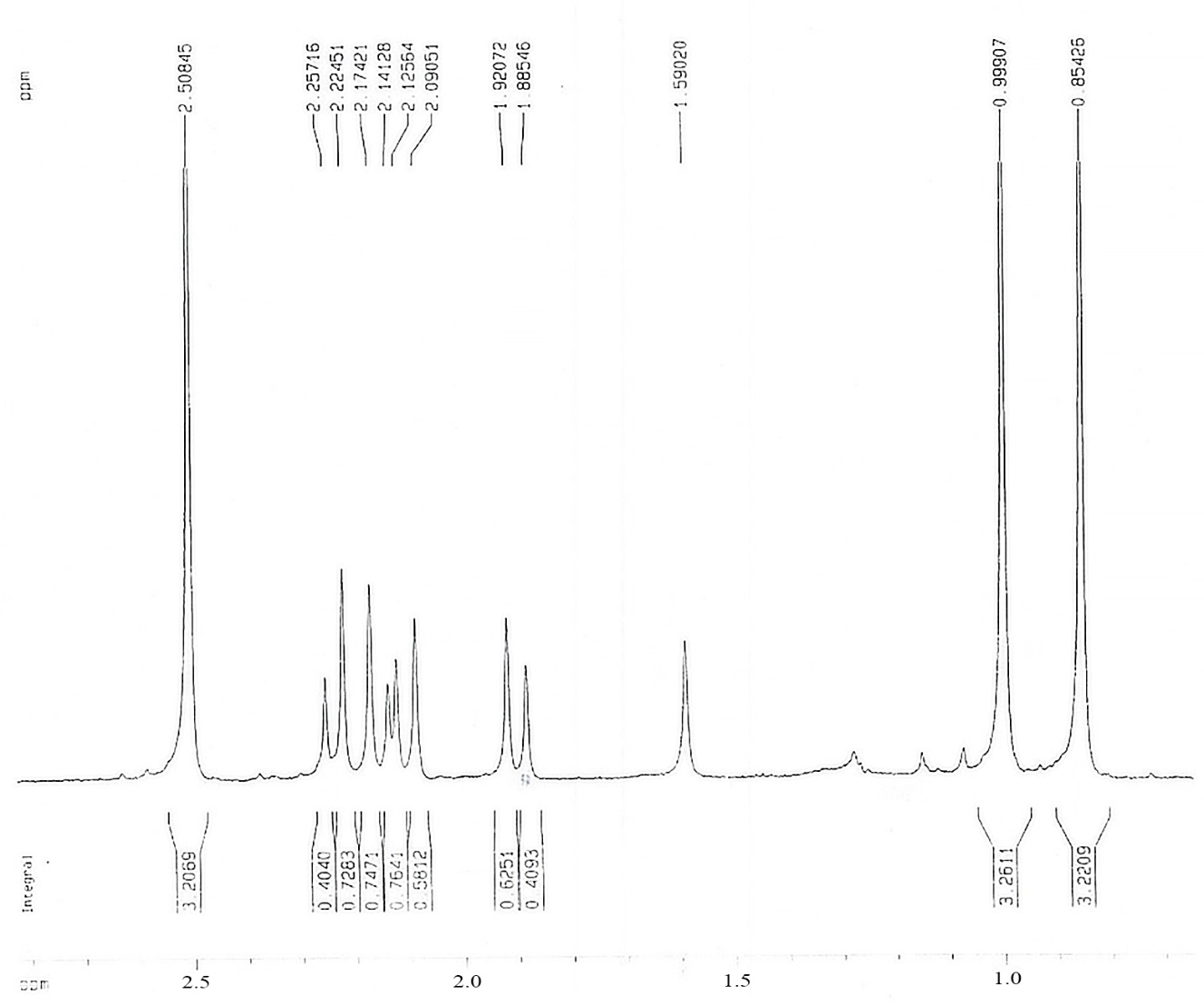


**Figure S7.** **^1^H NMR spectra of hydroquinoline.**
